# In situ quantification of ribosome number by electron tomography

**DOI:** 10.1111/jmi.13380

**Published:** 2025-01-15

**Authors:** Mounir El Hankouri, Marco Nousch, Aayush Poddar, Thomas Müller‐Reichert, Gunar Fabig

**Affiliations:** ^1^ Faculty of Medicine Carl Gustav Carus Experimental Center Technische Universität Dresden Dresden Germany; ^2^ Cluster of Excellence Physics of Life Technische Universität Dresden Dresden Germany; ^3^ Department of Developmental Genetics Institute of Biology Martin‐Luther‐Universität Halle‐Wittenberg Halle Germany; ^4^ Faculty of Medicine Carl Gustav Carus Core Facility Cellular Imaging Technische Universität Dresden Dresden Germany

**Keywords:** *C. elegans*, electron tomography, high‐pressure freezing, hTERT‐RPE‐1 cells, ribosome quantification, ribosomes

## Abstract

Ribosomes, discovered in 1955 by George Palade, were initially described as small cytoplasmic particles preferentially associated with the endoplasmic reticulum (ER). Over the years, extensive research has focused on both the structure and function of ribosomes. However, a fundamental question – how many ribosomes are present within whole cells – has remained largely unaddressed. In this study, we developed a microscopic method to quantify the total number of ribosomes in hTERT‐RPE‐1 cells and in nematode cells from various tissues of *Caenorhabditis elegans* hermaphrodites. Using electron tomography of high‐pressure frozen, freeze‐substituted and resin‐embedded samples, we determined that the ribosome number in hTERT‐RPE‐1 cells is in the same order of magnitude as biochemical measurements obtained via RNA capillary electrophoresis. As expected, control worms exhibited a higher number of ribosomes compared to RNA polymerase I A subunit (RPOA‐1)‐depleted worms in two out of three analysed tissue types. Our imaging‐based approach complements established biochemical methods by enabling direct quantification of ribosome numbers in specific samples. This method offers a powerful tool for advancing our understanding of ribosome localisation and distribution in cells and tissues across diverse model systems.

## LAY DESCRIPTION

The central dogma of cell biology stats that our genetic code (the DNA) within each cell is transcribed into an intermediate molecule (the RNA) which, in turn, is translated into the fundamental building blocks of life (the proteins). Translation is carried out by a small but very complex molecular machinery, called the ribosome. All cells on earth, from bacteria and archaea to those forming fungi, plants and animals rely on numerous ribosomes for protein production. Since their discovery in 1955 by George Palade we learned a lot about the function and structural composition of ribosomes. As ribosomes are very small (about 20–30 nm in diameter) they can only be visualised by electron microscopy which offers sufficient resolution. Up to now, however, the quantity and distribution of ribosomes in cells has not been explored in detail. Here we present a new method that uses a combination of 3D electron microscopy (electron tomography) and image analysis to measure the total number of ribosomes in a human tissue culture cell line. We also applied our new approach to a model organism, the roundworm *C. elegans*, and measured ribosome number in three different tissues of this nematode. Our new approach supplements already established biochemical analyses for tissue culture cells and will provide an additional systematic possibility to analyse ribosome number in other model organisms.

## 1 INTRODUCTION

Ribosomes are the cellular sites of protein synthesis. These macromolecular machines translate messenger RNA (mRNA) into polypeptide chains, thereby facilitating the expression of genes into functional proteins.^1^ While the key role of ribosomes in regulating cellular metabolism has been well established, prior research primarily focused on their translational activity. All ribosomes are composed of two subunits: a large and a small subunit. The large 60S subunit of the eukaryotic ribosome comprises three ribosomal RNA (rRNA) molecules (28S, 5.8S and 5S in mammals) and 46 proteins.^2^ The small 40S subunit consists of one rRNA chain (18S) and 33 proteins. Of the total 79 proteins in a ribosome, 32 lack structural homologs in bacterial or archaeal ribosomes, while those that do exhibit homologs contain significant eukaryote‐specific extensions.^3^ Apart from variability in certain rRNA expansion segments, all eukaryotic ribosomes – from yeast to human cells – are highly similar in structure.^4^ Despite significant advances in our understanding of ribosome function and structure in both prokaryotes and eukaryotes, a comprehensive analysis of ribosome abundance within cells remains insufficiently explored.

In practice, ribosome numbers can be analysed by either measuring the rRNA content (e.g., by capillary electrophoresis),^5^ or by quantifying ribosomal protein content either via Western blot or fluorescence intensity.^6^, ^7^ Both methods rely on indirect measurements, and an extrapolation of the ribosome numbers often yields inaccurate estimates, as is evident when comparing results obtained by these approaches.^8^ To complement these routinely used biochemical methods, we explored electron tomography as an imaging‐based approach for ribosome quantification. We established a semiautomated image analysis pipeline to segment and count ribosomes in immortalised human retinal pigment epithelial cells (hTERT‐RPE‐1). In addition, we quantified ribosome numbers in different regions of gonadal tissue and in somatic cells (vulva) of the nematode, *Caenorhabditis elegans*. For *C. elegans*, we further aimed to compare RNAi‐treated and control worms to detect anticipated differences in ribosome numbers. To this end, we targeted RPOA‐1, the RNA polymerase I A subunit, via RNAi feeding. Our results demonstrate that three‐dimensional (3D) transmission electron microscopy, particularly electron tomography, combined with our newly established semiautomated image analysis pipeline, can be employed to determine ribosome numbers in both single cells and cells organised within complex tissues. This 3D approach is well suited for robust quantification of ribosome numbers and is applicable to a broad range of model systems.

## 2 MATERIALS AND METHODS

### 2.1 Culture of hTERT‐RPE‐1 cells

HTERT‐RPE‐1 cells (ATCC CRL‐4000, Manassas, USA) were cultured in 175 cm^2^ cell culture flasks (Greiner Bio‐One GmbH, Frickenhausen, Germany) containing 10 mL of Dulbecco's Modified Eagle Medium (DMEM Glutamax; Gibco Fischer Scientific, Schwerte, Germany) with 10% (v/v) fetal bovine serum (FBS; Merck KGaA, Darmstadt, Germany) and 1% (v/v) 100× penicillin/streptomycin antibiotics (10,000 U/mL; Gibco Fischer Scientific, Schwerte, Germany). The cells were maintained in a humidified incubator at 37°C with 5% CO_2_ and passaged every 2–3 days upon reaching ∼70% confluency.

For passaging, the spent medium was discarded, and the cells were washed with prewarmed phosphate buffered saline (PBS, Gibco Fischer Scientific, Schwerte, Germany). After aspirating the PBS, the cells were treated with 0.25% trypsin‐EDTA (Gibco Fischer Scientific, Schwerte, Germany) for 3 min to facilitate detachment. To neutralise trypsin activity, FBS‐supplemented DMEM was added, followed by centrifugation of the cell suspension. The supernatant was aspirated, and the cell pellet was resuspended in fresh medium. Subsequently, one‐tenth of the suspension was transferred to a new flask containing fresh cell culture medium.

### 2.2 Cultivation of *C. elegans*

*C. elegans* (Bristol, N2) hermaphrodites were cultured on nematode growth medium (NGM) agar plates seeded with the *Escherichia coli* (*E. coli*) strain OP50 following standard protocols.^9^ For propagation, ten worms were transferred to fresh NGM plates every three to four days.

### 2.3 RNAi of RPOA‐1 in *C. elegans*

RNA‐mediated interference (RNAi) was performed by feeding bacteria expressing double‐stranded RNA (dsRNA) as described in published protocols.^10^, ^11^ The RNAi feeding construct targeting the *rpoa‐1* transcript was generated by cloning nucleotides 4073–5115 of the transcript *Y48E1A.1a.1* into the vector pL4440 (Addgene, Plasmid #1654), followed by transformation into *E. coli* strain HT115(DE3). Expression of dsRNA was induced by adding isopropyl β‐D‐thiogalactopyranoside (IPTG; Thermo Fisher Scientific GmbH, Dreieich, Germany) to a final concentration of 1 mM, following established protocols.^12^ Approximately 40 L4‐larval stage animals were transferred to RNAi‐expressing bacteria on NGM plates for 24 h at 25°C.

The primers used for amplifying nucleotides 4073–5115 from *Y48E1A.1a.1* were:

Forward primer: TGCACTTGTTCGTCGATCCA;
Reverse primer: GAGAAGACGGTCCTGCAACA.

The genome of wild‐type *C. elegans* (N2, Bristol) was used as a template for the amplification of the nucleotides 4073–5115 from the transcript *Y48E1A.1a.1*. As a control, the empty vector pL4440 was transformed into the same bacterial strain, *E. coli* HT115(DE3), and treated similarly with IPTG as the dsRNA‐expressing bacteria.

### 2.4 High‐pressure freezing and freeze substitution

The hTERT‐RPE‐1 cells were cultured and attached to sapphire discs as previously described.^13^ Briefly, a ‘sandwich’ for high‐pressure freezing was assembled by gently placing a sapphire disc (Wohlwend, article no. 500) with attached cells upside down onto an aluminium planchette with a cavity depth of 0.04 mm (Wohlwend, article no. 737). Cell culture medium supplemented with additional 10% (w/v) bovine serum albumin (BSA; Carl Roth, Karlsruhe, Germany) was used as a cryoprotectant. Samples were cryo‐immobilised under ∼2000 bar of pressure and a cooling rate of ∼20,000°C/sec using a Compact 03 high‐pressure freezer (Wohlwend, Switzerland), as described previously.^14^, ^15^

For the cryo‐immobilisation of worms, sample holders (type‐A aluminium planchettes; Wohlwend, article no. 241) were precoated with 1‐hexadecene (Merck KGaA, Darmstadt, Germany) and subsequently filled with M9 buffer containing 10% (w/v) polyvinylpyrrolidone (PVP; MW 10,000; Merck KGaA, Darmstadt, Germany) as the cryoprotectant during high‐pressure freezing.^15^, ^16^, ^17^ Approximately five gravid adult hermaphrodites (24 h post‐L4 stage) were transferred into the 100 µm indentation of each type‐A planchette for each round of freezing. The specimen holders were carefully closed by placing the flat side of a type‐B aluminium planchette (Wohlwend, article no. 242) on top of the type‐A planchette. Following high‐pressure freezing, the closed sample holders were stored in liquid nitrogen until further use.

During freeze substitution, sample holders containing either hTERT‐RPE‐1 cells or worms were opened under liquid nitrogen using a syringe needle. Sapphire discs with attached frozen hTERT‐RPE‐1 cells and type‐A planchettes containing worms were transferred to cryovials filled with anhydrous acetone supplemented with 1% (w/v) osmium tetroxide (EMS, Hatfield, USA) and 0.1% (w/v) uranyl acetate (Polysciences, Warrington, UK) as described previously.^18^ Freeze substitution was performed using an automatic freeze substitution system (AFS2, Leica Microsystems, Vienna, Austria). Samples were maintained at −90°C for 1 h, warmed to −30°C at a rate of 5°C/h, held at −30°C for 5 h, and then warmed to 0°C in 5°C/h increments.^15^

### 2.5 Resin embedding, re‐mounting and ultramicrotomy

Immediately after freeze‐substitution, hTERT‐RPE‐1 and worm samples were washed three times with pure anhydrous acetone and subsequently infiltrated with Epon/Araldite resin (EMS, Hatfield, USA) at increasing concentrations (resin:acetone ratios of 1:3, 1:1, 3:1, and finally pure resin), with each step lasting 2 h at room temperature.^18^ Samples were incubated overnight in pure resin and then again for 4 h. hTERT‐RPE‐1 cells were mounted in flow‐through chambers as previously described.^14^ Worms were thin‐layer embedded between two Teflon^®^‐coated glass slides, following established protocols.^18^ The samples were polymerised at 60°C for 48 h. Resin blocks containing hTERT‐RPE‐1 cells and thin‐layer embedded worms, which were re‐mounted on dummy blocks,^18^ were serially sectioned using an ultramicrotome (EM UC6, Leica Microsystems, Austria). Ribbons of serial sections (300 nm thick) were collected on Formvar‐coated copper slot grids and post‐stained with 2% (w/v) uranyl acetate in 70% (v/v) methanol, followed by 0.4% (w/v) lead citrate in double‐distilled water.^14^ Finally, both sides of the samples were coated with 20 nm colloidal gold (BBI, Crumlin, UK), which served as fiducial markers for subsequent tomographic reconstruction.^13^

### 2.6 Electron tomography and 3D reconstruction

The serial semithick sections were prescreened at low magnification by using a transmission electron microscope (Morgagni, Thermo Fisher Scientific, USA) operated at 80 kV and equipped with a 2k × 2k CCD camera (Veleta, EMSIS, Germany), enabling the identification of hTERT‐RPE‐1 cells in interphase. Distal and pachytene regions and also vulva cells in the *C. elegans* samples were identified. Sections were then transferred to a transmission electron microscope (TECNAI F30, Thermo Fisher Scientific, USA) operated at 300 kV and equipped with a 4k × 4k sCMOS camera (OneView, Gatan, USA). Tilt series were acquired using a dual‐axis specimen holder (Type 2040, Fischione Instruments, USA), spanning angles from −60° to +60° in 1° increments at a magnification of 4700× and a pixel size of 2.572 nm, with the SerialEM software package.^19^, ^20^ After rotating the grids 90° in the X/Y‐plane, a second tilt series of the selected positions was acquired using identical microscope settings.^21^ The tomographic A‐ and B‐stacks for each region of interest were reconstructed, combined, flattened, and trimmed in Z using the IMOD software package.^22^, ^23^

### 2.7 Automatic segmentation of ribosomes in electron tomograms

Plastic‐embedded samples collapse in the electron beam during the acquisition of tomograms.^24^, ^25^ To account for this sample collapse, a shrinkage factor must be calculated.^26^ As previously determined for serial sections, this factor is given by the ratio between the calculated thickness (as obtained after tomographic reconstruction of stitched serial sections) and the initial thickness (as obtained from the ultramicrotome settings and number of sections). This factor is then multiplied with the pixel size in the Z‐dimension of each tomogram. Analysis of 77 stitched data sets generated in our laboratory (see Data S1), yielded a mean shrinkage factor of 1.575 for samples embedded in Epon/Araldite. This factor was subsequently applied to the data sets presented here.

The MRC‐files resulting from the reconstruction process were then converted to TIFF‐files (see Figure S1A) and further processed with the Fiji software package (version 1.8.0_322).^27^ This program facilitated the selection of regions of interest (ROIs) and enabled cropping of selected subregions from each tomogram devoid of any organelles such as mitochondria. All selected regions and the accompanying tomograms are listed in Table 1 and can be accessed via a publicly available OMERO link.

**TABLE 1:** Overview of all created electron tomographic data sets of hTERT‐RPE‐1 cells and *C. elegans* used for detailed analysis.

Sample	Condition	Data set name
hTERT‐RPE‐1 cell, interphase	Wild type	T0611_CRL‐4000_WT_Block01_Cell01_TOMO_Interphase01
hTERT‐RPE‐1 cell, interphase	Wild type	T0661_CRL‐4000_WT_Block06_Cell03_TOMO_Interphase02
hTERT‐RPE‐1 cell, interphase	Wild type	T0661_CRL‐4000_WT_Block06_Cell01_TOMO_Interphase03
hTERT‐RPE‐1 cell, interphase	Wild type	T0661_CRL‐4000_WT_Block06_Cell02_TOMO_Interphase04
hTERT‐RPE‐1 cell, interphase	Wild type	T0661_CRL‐4000_WT_Block02_Cell01_TOMO_Interphase05
hTERT‐RPE‐1 cell, interphase	Wild type	T0661_CRL‐4000_WT_Block01_Cell01_TOMO_Interphase06
*C. elegans*, gonad distal	Control (RNAi)	T0672_N2_controlRNAi_DistalGonad_Worm02_TOMO_Gonad01
*C. elegans*, gonad distal	Control (RNAi)	T0672_N2_controlRNAi_DistalGonad_Worm08_TOMO_Gonad02
*C. elegans*, gonad distal	Control (RNAi)	T0672_N2_controlRNAi_DistalGonad_Worm09_TOMO_Gonad03
*C. elegans*, gonad distal	Control (RNAi)	T0672_N2_controlRNAi_DistalGonad_Worm04_TOMO_Gonad04
*C. elegans*, gonad pachytene	Control (RNAi)	T0672_N2_controlRNAi_PachyteneGonad_Worm04_TOMO_Gonad01
*C. elegans*, gonad pachytene	Control (RNAi)	T0672_N2_controlRNAi_PachyteneGonad_Worm07_TOMO_Gonad02
*C. elegans*, gonad pachytene	Control (RNAi)	T0672_N2_controlRNAi_PachyteneGonad_Worm09_TOMO_Gonad03
*C. elegans*, gonad pachytene	Control (RNAi)	T0672_N2_controlRNAi_PachyteneGonad_Worm06_TOMO_Gonad04
*C. elegans*, vulva cell	Control (RNAi)	T0672_N2_controlRNAi_Vulvacell_Worm06_TOMO_Interphase01
*C. elegans*, vulva cell	Control (RNAi)	T0672_N2_controlRNAi_Vulvacell_Worm07_TOMO_Interphase02
*C. elegans*, vulva cell	Control (RNAi)	T0672_N2_controlRNAi_Vulvacell_Worm09_TOMO_Interphase03
*C. elegans*, vulva cell	Control (RNAi)	T0672_N2_controlRNAi_Vulvacell_Worm04_TOMO_Interphase04
*C. elegans*, gonad distal	*rpoa‐1(RNAi)*	T0675_N2_RPOA‐1RNAi_DistalGonad_Worm05_TOMO_Gonad01
*C. elegans*, gonad distal	*rpoa‐1(RNAi)*	T0671_N2_RPOA‐1RNAi_DistalGonad_Worm11_TOMO_Gonad02
*C. elegans*, gonad distal	*rpoa‐1(RNAi)*	T0671_N2_RPOA‐1RNAi_DistalGonad_Worm13_TOMO_Gonad03
*C. elegans*, gonad distal	*rpoa‐1(RNAi)*	T0679_N2_RPOA‐1RNAi_DistalGonad_Worm04_TOMO_Gonad04
*C. elegans*, gonad pachytene	*rpoa‐1(RNAi)*	T0679_N2_RPOA‐1RNAi_PachyteneGonad_Worm10_TOMO_Gonad01
*C. elegans*, gonad pachytene	*rpoa‐1(RNAi)*	T0671_N2_RPOA‐1RNAi_PachyteneGonad_Worm11_TOMO_Gonad02
*C. elegans*, gonad pachytene	*rpoa‐1(RNAi)*	T0671_N2_RPOA‐1RNAi_PachyteneGonad_Worm13_TOMO_Gonad03
*C. elegans*, vulva cell	*rpoa‐1(RNAi)*	T0675_N2_RPOA‐1RNAi_Vulvacell_Worm05_TOMO_Interphase01
*C. elegans*, vulva cell	*rpoa‐1(RNAi)*	T0671_N2_RPOA‐1RNAi_Vulvacell_Worm08_TOMO_Interphase02
*C. elegans*, vulva cell	*rpoa‐1(RNAi)*	T0671_N2_RPOA‐1RNAi_Vulvacell_Worm11_TOMO_Interphase03
*C. elegans*, overview	Wild type	T0596_N2_wild‐type_Overview_WormH_TEM_Gonad
*C. elegans*, overview	*rpoa‐1(RNAi)*	T0679_N2_RPOA‐1RNAi_Overview_Worm05_TEM_Gonad

The TIFF‐files were then imported into Arivis Vision4D 3.4. After setting the pixel size to 2.572 nm, the following procedure was performed: All planes of the cropped image were selected (ROI: custom → planes → all → scaling 100%) as input. The images were then inverted to detect the stained ribosomes as local maxima (see Figure S1B) and a denoising algorithm (method: median filter → diameter  =  7.72 nm → Rank  =  50%) was applied to remove background noise (see Figure S1C). The resulting images were stored. Subsequently, the segmentation function ‘Blob Finder’ was utilised to segment the ribosomes (see Figure S1D). The specific parameters for the analysed images were: diameter, 30 nm; probability threshold, 30%; and split sensitivity, 70%. Since eukaryotic ribosomes are reported to have a diameter of 20–30 nm,^28^, ^29^ the maximum size in the tool was set to 30 nm. According to the software manufacturer (pers. comm.), the tool can detect particles ranging in size from 1 and 30 nm. Following execution of the Blob Finder algorithm, objects were detected, and a touching edge filter (input: Blob Finder → Edges to exclude: Z top section, Z bottom section → Output: Touching edge filter) was applied (see Figure S1E). After this initial filtering process, a second filter was applied (see Figure S1F) to remove flat segments: segment feature filter (input: Touching edge filter → Plane: count → > 1 → Outputs: segment feature filter). Finally, all the objects and inputs were stored, and the object table from the segment feature filter output was exported as an. xlsx file for further analysis.

To reduce the number of false‐positive segmentations, upper and lower limits for the sizes of detected particles were defined. Considering that a eukaryotic ribosome has an approximate maximum diameter of 30 nm, and is roughly spherical, its volume should be approximately 14,137 nm^3^ (4/3 × π × 15 × 15 × 15). With a voxel volume of approximately 17 nm^3^ (2.572 × 2.572 × 2.572) this calculation yields a maximum voxel count of about 830 per ribosome. Based on this calculation, objects larger than 830 voxels were excluded.

For the lower limit, the assumption of a ribosome diameter of 20 nm was applied. Taking the average shrinkage factor in Z into account, then the volume would approximately be 2660 nm^3^ (4/3 × π × 10 × 10 × 10/1.575). This translated to a voxel count of 156. As a ribosome are unlikely to be perfectly spherical, this number was rounded down to 150 voxels. Objects with fewer than 150 voxels were excluded. However, this approach resulted in the exclusion of many objects that experienced electron microscopists could identify as ribosomes. To address this, the voxel count exclusion criterion was lowered stepwise (see Figures S2 and S3). Thresholds of 25, 50, and 100 voxels were determined empirically, which yielded robust ribosome detections in hTERT‐RPE‐1 cells and *C. elegans* samples.

In tomography‐based analyses, the reported ribosome number depended on the selected voxel count threshold. The observed discrepancy between the measured and the theoretical ribosome size is likely due to shrinkage during sample preparation.

### 2.8 Calculation of the total number of ribosomes in hTERT‐RPE‐1 cells

After cropping five subregions per tomogram for ribosome segmentation, the size of the respective subvolumes was determined. The number of objects per subregion volume was calculated per cubic micrometre and divided by the shrinkage factor. The resulting measurement represents the normalised, shrinkage‐corrected total number of ribosomes per cubic micrometre of cytoplasmic volume. An identical calculation was applied to five cropped subregions per tomogram in both distal and pachytene regions of the gonad, as well as in somatic cells of the vulva in the control group of worms. A similar analysis was conducted for *rpoa‐1(RNAi)* worms.

### 2.9 Determination of hTERT‐RPE‐1 cell size

The volume of hTERT‐RPE‐1 cells was determined using cultured cells (up to passage no. 4) treated with 0.25% trypsin followed by gentle pipetting to break cell‐cell contacts. Subsequently, cells were incubated in a cell imaging dish for 2–4 h to allow attachment, staining, and fixation, resulting in rounded cells devoid of cell‐cell contacts. Staining involved use of MemBrite^®^ Fix Pre‐Staining Solution 1× (Biotium, Fremont, USA) followed by MemBrite^®^ Fix 543/560 dye solution 1× (Biotium, Fremont, USA), with subsequent fixation in 100% ice‐cold methanol. The nuclei were stained with DAPI (Merck KGaA, Darmstadt, Germany). Fixed and stained cells were analysed using a Leica TCS SP5 laser scanning confocal microscope (Leica Microsystems, Wetzlar, Germany) equipped with a 40×/1.25 HCX PL APO lambda blue oil objective. Initially, cells were located through the ocular lenses before transitioning to laser scanning for image acquisition. For DAPI staining, samples were illuminated using a 405 nm laser, with emission signals collected within the spectral range of 415–480 nm. For MemBrite® staining, samples were illuminated using a 543 nm laser and signals were collected within the spectral range of 550–700 nm. Images were acquired in a XY‐scan field of 512 × 512 pixels and with a Z‐dimension sufficient to capture the entire cell volume (40–50 µm).

The images obtained by laser scanning confocal microscopy underwent initial processing using Fiji. This process included merging the fluorescence channels (DAPI and MemBrite^®^ dye) for each image, preselecting cells of interest, and cropping them for individual analysis. The cropped images were saved as TIFF‐files, imported into Arivis Vision4D 3.5, and further analysed. In the Arivis analysis pipeline, the bilateral denoising algorithm (diameter 5 µm, sensitivity 51) and automatic background correction algorithm (blur diameter 150 µm, preserve bright) were applied. The entire image stack of the membrane channel was used for membrane segmentation via the magic wand tool, enabling precise delineation of cell boundaries across all planes. Segmented objects were manually merged into 3D objects in the object table to facilitate analysis of cell volume. Subsequently, the same methodology was applied to segment nuclei in the DAPI channel and determine their volumes. For each of the ten interphase cells, the volume of the nucleus (devoid of ribosomes) was subtracted from the volume of the entire cell. Additionally, 10% of this resulting volume was subtracted to account for other organelles.^30^ The final average cytoplasmic volume was used for subsequent calculations.

As treatment with cold pure methanol could potentially lead to dehydration artefacts such as shrinkage, the volume of live and fixed cells was compared (*n* = 13). For this, hTERT‐RPE‐1 cells were prepared as described and imaged using phase contrast optics before and after treatment with pure cold methanol. For this, a Zeiss Observer microscope (Carl Zeiss AG, Oberkochen, Germany) equipped with a Plan‐Apochromat 40×/0.95 objective, and an Axiocam 506 mono camera, was used. The collected images were then analysed using the Fiji software package (version 1.54f) in the following way: after selecting the cells of interest in the ROI manager, selected regions were cropped and denoised using the ‘smooth’ function. After manually adjusting the threshold to fit the cell boundaries, the images were converted into binary images and the function ‘fill holes’ was applied. Then the cells of interest were segmented, cellular area was measured and the measurements before and after methanol treatment were compared using Welch's two‐sided *t*‐test within the RStudio software package (version 4.4.1).

### 2.10 High‐resolution automated electrophoresis of RNA samples

The hTERT‐RPE‐1 cells, at approximately 80% confluency, were counted by transferring 2 × 5 µL aliquots of cell suspension into a haemocytometer. These initial cell cultures were diluted to prepare triplicates of suspensions each containing either one or two million cells in 15 mL conical tubes. The tubes were centrifuged at 200 rcf for 6 min to sediment the cells. The medium was subsequently removed, and the tubes containing the pellets were immediately placed in liquid nitrogen to preserve cellular integrity and prevent RNA degradation. Total RNA was extracted using a previously published phenol:chloroform protocol.^31^ The extracted RNA was eluted in 50 µL of RNase‐free water and diluted 1:50 in RNase‐free water to achieve a concentration suitable for reliable measurement.

### 2.11 Extrapolation of number of ribosomes from RNA capillary electrophoresis data

The quality and quantity of total RNA, including the 18S and 28S ribosomal RNA fragments extracted from the two cell concentrations of hTERT‐RPE‐1 (one million and two million cells), were assessed using an Agilent 2100 Bioanalyzer (Agilent, Santa Clara, USA) and the Agilent RNA 6000 Nano Kit (Agilent, Santa Clara, USA) in accordance with the manufacturer's protocol (bit.ly/agilentrna6000nano). In a successful run, the software generates a data plot of size/migration time versus fluorescence intensity. Peaks are automatically identified, with their values tabulated by peak ID, including ribosomal RNA fragments (see Data S2).

An RNA ladder containing a mixture of RNA at known concentrations was initially analysed. To calculate the total RNA concentration, the area under the entire RNA electropherogram curve was automatically determined by the software. The ladder, which provided the concentration‐to‐area ratio, enabled the software to transform area values into concentration values. For the 18S and 28S rRNA fragments, the areas under their respective peaks were automatically determined by the software, with a threshold or baseline set under the peaks for measurement.

To account for potential inaccuracies in automated thresholding or baselining, manual adjustments were performed, as recommended in the manufacturer's user guide.^32^ The areas under the 18S and 28S rRNA peaks were then converted to concentrations. The RNA integrity number (RIN), which reflects rRNA quality, was calculated automatically and displayed both in the results subtab and below the gel‐like image. The RIN values generated by the Agilent 2100 Bioanalyzer indicate rRNA quality, with values ranging from 7 and 10 denoting good quality.^33^ Only samples with a RIN > 9 were included in further calculations.

To estimate the number of ribosomes per hTERT‐RPE‐1 cell, the quantities of the 18S and 28S rRNA subunits were calculated using the equation provided below (see also Data S3). The mean of the total number of these two subunits was then determined. As a single ribosome is composed of one 18S and one 28S subunit, the mean number of these subunits per cell was used to derive the final number of ribosomes per hTERT‐RPE‐1 cell.

$$\begin{array}{ccc} & & \mathrm{No}.\;\mathrm{of}\;18S\;\mathrm{or}\;28S\;\mathrm{subunits}\;\mathrm{per}\;\mathrm{cell}\;\\ & & \;=\frac{\frac{\mathrm{rRNA}\;\mathrm{concentration}\;\times \;\mathrm{elution}\;\mathrm{volume}\;\times \;\mathrm{Avogadro}\;\mathrm{constant}}{\mathrm{molecular}\;\mathrm{mass}\;\mathrm{of}\;18S\;\mathrm{or}\;28S\;\mathrm{subunit}}}{\mathrm{Number}\;\mathrm{of}\;\mathrm{cells}} \end{array}$$

## 3 RESULTS

### 3.1 Concentration of ribosomes in subvolumes of hTERT‐RPE‐1 cells

**FIGURE 1:**
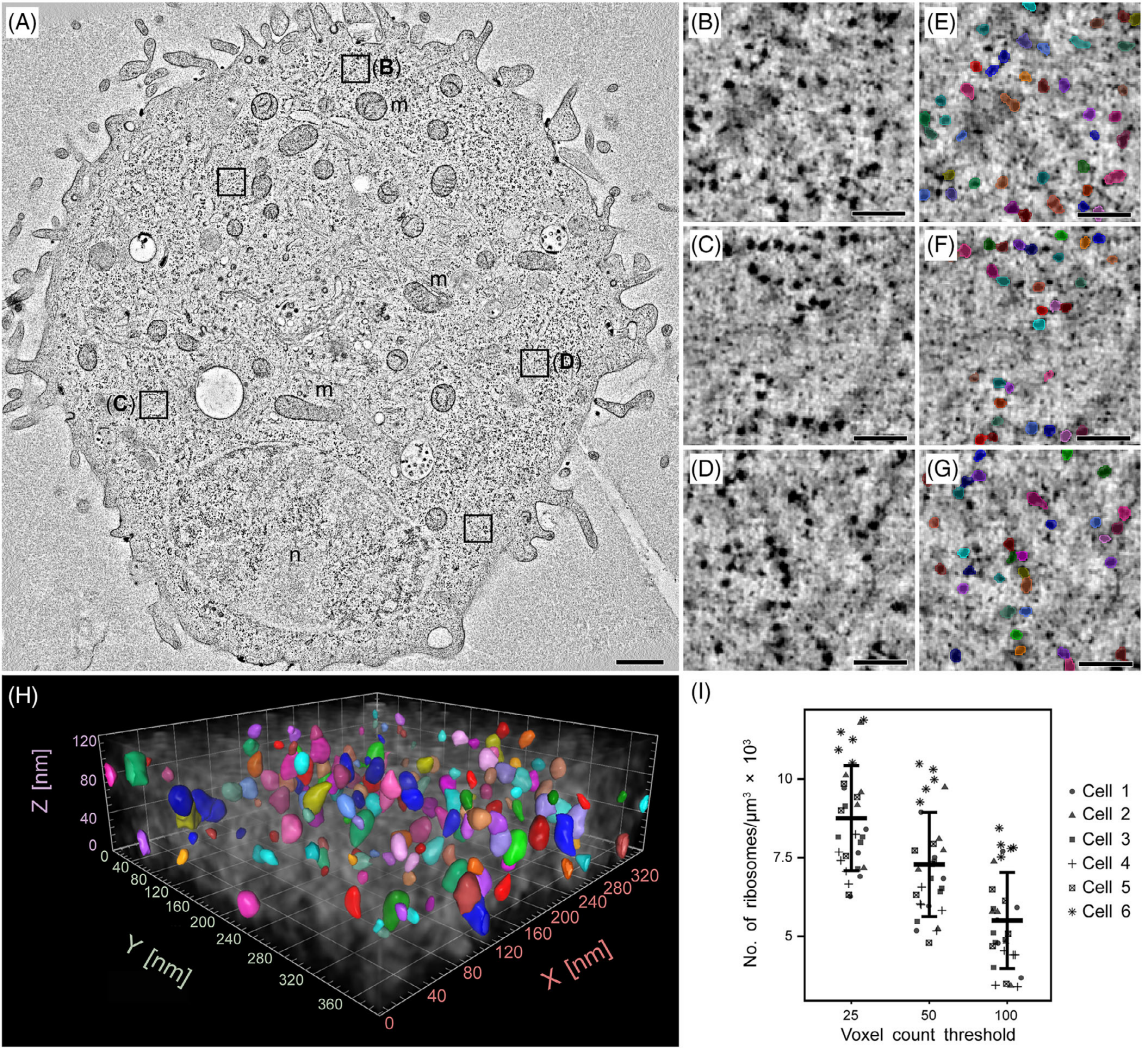
**Quantitative analysis of ribosome number in hTERT‐RPE‐1 cells**. (**A**) Tomographic slice showing an interphase cell with five selected regions cropped out for detailed data analysis (black squares). The nucleus (n) of this cell and numerous mitochondria (m) are visible. Scale bar, 1 µm. (**B–D**) Three out of five segmented and analysed cytoplasmic regions as indicated in A. Scale bars, 100 nm. (**E–G**) Automatic segmentation of ribosomes (coloured overlay) in the three selected regions as shown in B–D, using a voxel count threshold of 25. Scale bars, 100 nm. (**H**) Volumetric view showing the ribosomes in the fully segmented Z‐stack of the region as shown in C and F. (**I**) Quantification of ribosome number in five selected subregions of six different hTERT‐RPE‐1 cells. The mean and standard deviation of the ribosome numbers per cubic micrometre over all six samples is given for three different voxel count thresholds. For the individual plots, the different symbols represent the individual measurements from the five subregions in the six cells as indicated by the legend.

To demonstrate, in a proof‐of‐concept study, that an image‐based approach can enable robust quantification of ribosome numbers in human tissue culture cells, the number of ribosomes was determined in defined cytoplasmic subvolumes of high‐pressure frozen, freeze‐substituted, and resin‐embedded hTERT‐RPE‐1 cells using electron tomography. In total, six tomograms were generated from six different hTERT‐RPE‐1 cells in interphase, and five subvolumes were selected from each tomogram for further analysis (Figure 1A–D). Subvolumes containing only ribosomes and excluding other organelles, such as mitochondria or large vesicles, were selected.

Correcting for background noise and excluding particles larger than 30 nm, ribosomes were automatically segmented in these subvolumes using the Arivis Vision4D software package (Figure 1E–G). This procedure was followed by the application of three different voxel count thresholds (25, 50, 100, as described in the Section 2.7). Visual inspection confirmed the accuracy of this automatic segmentation procedure. The ribosome count in the given tomographic volumes was calculated. Electron beam exposure induced beam‐related shrinkage in the thickness of the sectioned sample resulting in a reduced Z‐dimension (Figure 1H). This reduction was corrected by multiplying with the determined mean shrinkage factor (see Section 2.8 for details). After this correction, the average number of ribosomes per cubic micrometre for a given voxel count threshold was determined.

The analysis resulted in mean ribosome concentrations of 8754 ± 1644, 7282 ± 1628, and 5505 ± 1505 ribosomes per cubic micrometre for voxel count thresholds of 25, 50, and 100, respectively (Figure 1I Data S4). Thus, the developed approach enables quantification of ribosome numbers within a cytoplasmic subvolume of hTERT‐RPE‐1 cells while providing an empirically derived selection criterion.

### 3.2 Total number of ribosomes in hTERT‐RPE‐1 cells via combination of ET and light microscopy approach

**FIGURE 2:**
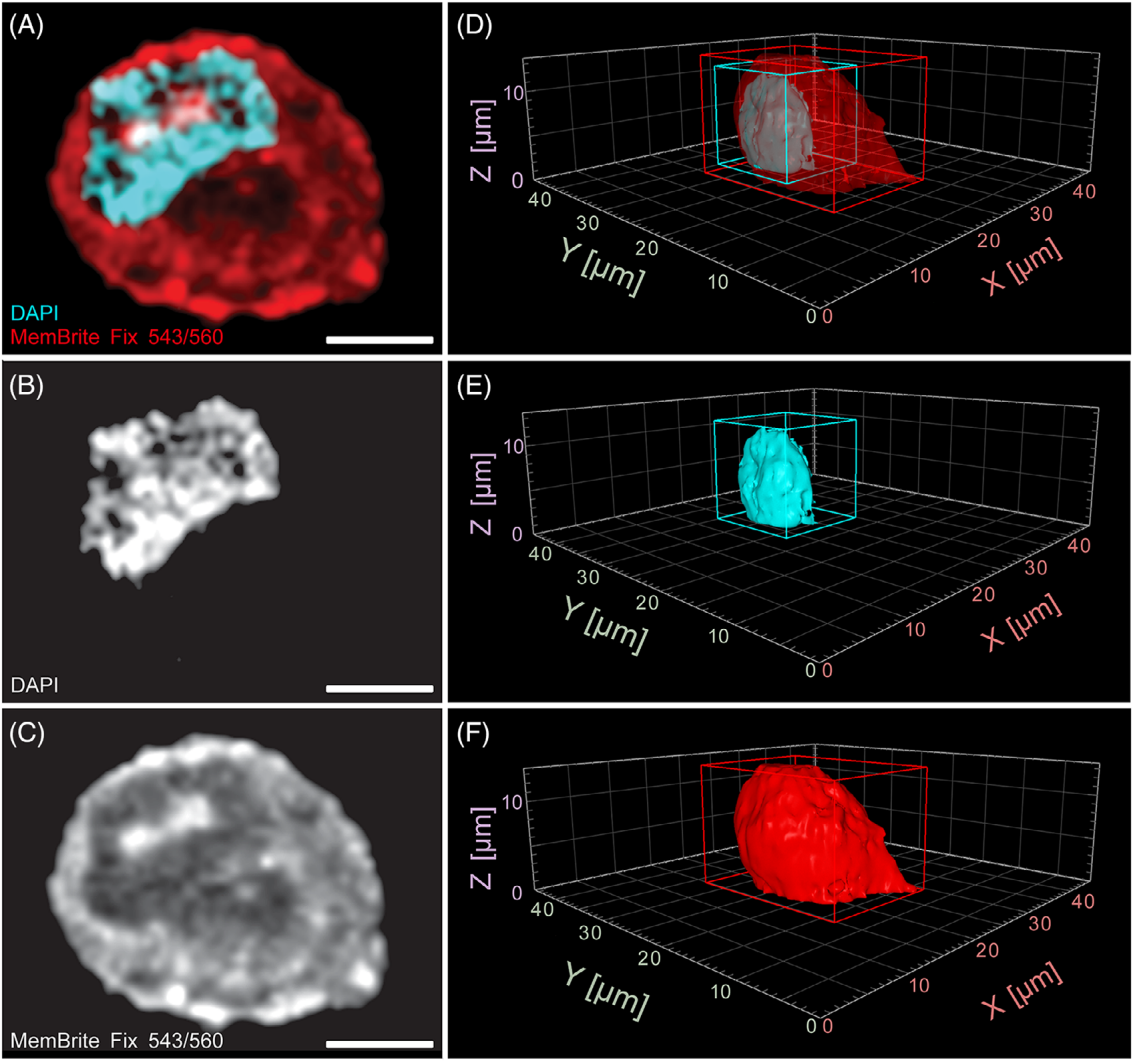
**Volume analysis of hTERT‐RPE‐1 cells by confocal light microscopy**. (**A**) Single slice from an acquired confocal Z‐stack of a fixed hTERT‐RPE‐1 cell in interphase. The cell membrane and the cytoplasm are shown in red, the nucleus in cyan. (**B**) Single slice showing only the nucleus in the confocal Z‐stack as illustrated in A. (**C**) Single slice showing only the membrane and cytoplasm in the confocal Z‐stack as shown in (A). (**D–F**) Volumetric view of the 3D‐segmented cell, nucleus and cytoplasm as shown in A–C. Scale bars, 5 µm.

Next, the aim was to quantify the total number of ribosomes in entire hTERT‐RPE‐1 cells. To achieve this, it was necessary to determine the total cytoplasmic volume of this mammalian cell type. This was accomplished by measuring the total volume of hTERT‐RPE‐1 cells using confocal fluorescence microscopy and subsequently subtracting the nuclear volume from the total cellular volume. MemBrite^®^ was used to stain the cell membrane, and DAPI was used to stain the nucleus (Figure 2A–C). The volumes of ten hTERT‐RPE‐1 cells and their nuclei were then determined by 3D segmentation using Arivis Vision4D 3.5 (Figure 2D–F).

This analysis revealed that the hTERT‐RPE‐1 cells, on average, had a total volume of 2638.9 ± 925.9 µm^3^ and a nuclear volume of 652.9 ± 137.9 µm^3^ during interphase. Consequently, the calculated average cytoplasmic volume was 1986.0 ± 855.4 µm^3^. Considering that approximately 8% of the total cell volume in RPE cells is occupied by mitochondria,^30^ as reported previously, and accounting for other smaller organelles, the cytoplasmic volume was reduced by 10%. This adjustment resulted in an average cytoplasmic volume of 1787.4 ± 769.8 µm^3^ (see Data S5).

Consistent with previously published findings,^34^ the methanol‐treated cells exhibited no significant difference in cell area compared to untreated cells (see Data S6).

The calculated cytoplasmic volume was then multiplied by the number of ribosomes per cubic micrometre, as determined by electron tomography. This resulted in mean ribosome counts per hTERT‐RPE‐1 cell of 15.6 × 10^6^ ± 1.3 × 10^6^, 13.0 × 10^6^ ± 1.3 × 10^6^, and 9.8 × 10^6^ ± 1.2 × 10^6^ for voxel count thresholds of 25, 50, and 100, respectively (see Data S5).

By integrating electron and light microscopic analyses, the total ribosome count in the hTERT‐RPE‐1 cell line was determined across a range of distinct voxel count thresholds.

### 3.3 Biochemical quantification of ribosomes in hTERT‐RPE‐1 cells

**FIGURE 3:**
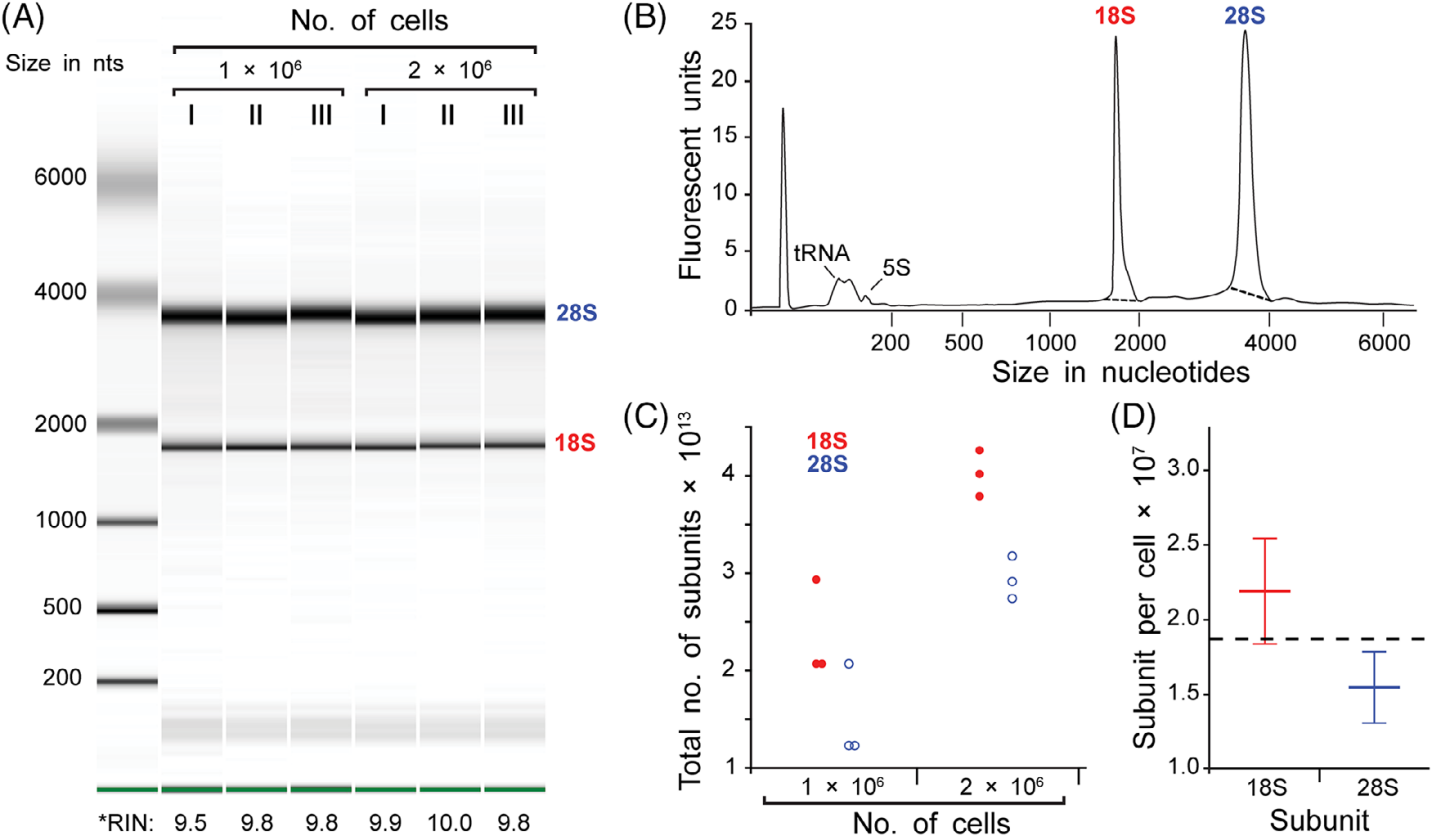
**Biochemical determination of ribosome number in hTERT‐RPE‐1 cells**. (**A**) Capillary electrophoresis of extracted total RNA. Green bands at the bottom represent the marker, the bands below 200 nucleotides represent tRNA and 5S RNA, the bands below 2000 nucleotides represent the 18S rRNA, the bands below 4000 nucleotides represent the 28S rRNA. RNA integrity number (RIN) indicates the RNA quality and is given below the plot. (**B**) Example electropherogram of the capillary electrophoresis of the total RNA extracted from one sample (lane II in A). (**C**) Graph showing the extrapolated results from the capillary electrophoresis approach estimating the number of subunits of 18S (red dot) and 28S (blue ring) subunits in one million (left) and two million (right) cells. (**D**) Graph showing the mean number of 18S and 28S subunits per single hTERT‐RPE‐1 cell. Error bars indicate the standard deviation. The dashed line indicates the mean number of subunits per cell. See also Data S2.

The total number of ribosomes in hTERT‐RPE‐1 cells was also determined using a biochemical approach, enabling a direct comparison of biochemically and microscopically derived EM results on ribosome number. Capillary electrophoresis was performed on extracted total RNA from both one and two million hTERT‐RPE‐1 cells, each in triplicates (Figure 3A). Using two different cell concentrations, the study aimed to validate the results across different scales, confirming that the quantification method is robust and independent of cell concentrations. For this analysis, only RNA samples with an RNA Integrity Number (RIN) > 9 were considered, resulting in clean electropherograms with well‐defined 18S and 28S rRNA fragment peaks and without fluctuations in the fluorescence signal (Figure 3B). Using manually adjusted thresholds (indicated by dashed lines in Figure 3B and coloured lines in Data S2), automated measurements of the areas under the 18S and 28S rRNA fragment peaks were performed to calculate the concentration of both rRNA subunits for each sample. These concentrations were then converted to the number of subunits (Figure 3C) using the equation described in the Section 2 (see Data S3).

The total number of 18S and 28S subunits extracted from two million cells was approximately twice as high as that obtained from the analysis of one million cells (Figure 3C, left and right sides), thus validating the robustness of this biochemical approach. Next, the number of ribosomes per hTERT‐RPE‐1 cell was determined. For this, the number of subunits per cell was calculated as the average number of 18S and 28S subunits from one million cells and two million cells, each divided by the respective number of cells (Figure 3D). Given that the total number of 18S and 28S subunits corresponds to the total number of ribosomes (since the two subunits form one ribosome^4^), the biochemical approach showed an average of 18.7 × 10^6^ ± 4.4 × 10^6^ ribosomes per single hTERT‐RPE‐1 cell (Figure 3D Data S3). With the application of three different voxel count thresholds, a range in ribosome numbers was determined. The result from the biochemical quantification closely matches the electron tomography data, which showed 15.6 × 10^6^ ± 1.3 × 10^6^ ribosomes using a voxel count threshold of 25.

### 3.4 Quantification of ribosome numbers in *C. elegans* samples

**FIGURE 4:**
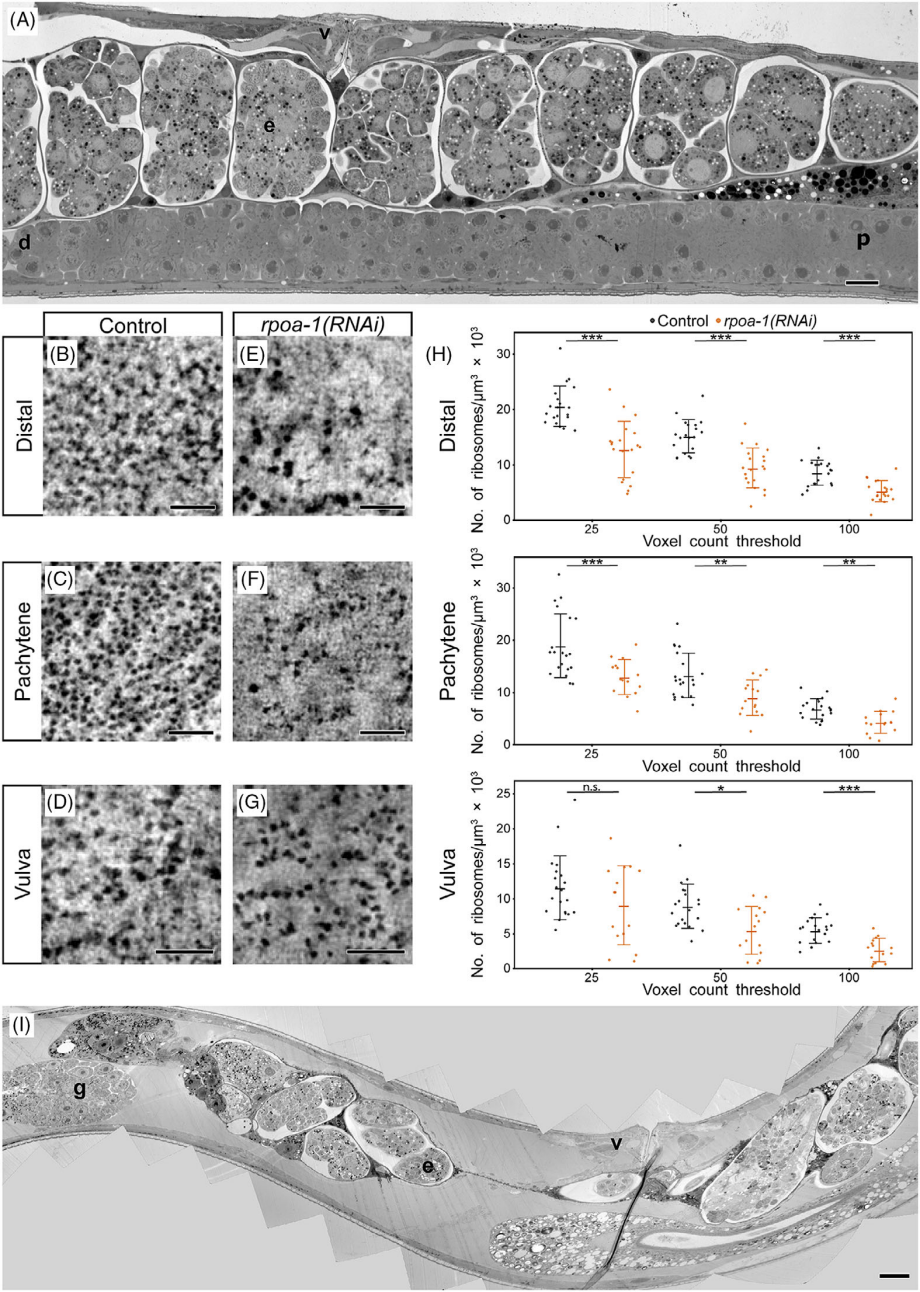
**Ribosome number in germ cells and somatic cells of *C. elegans***. (**A**) Overview TEM image showing the morphology of a wild‐type hermaphrodite with mitotic germ cells in the distal region of the gonad (d), meiotic cells in the pachytene stage (p, middle region of the gonad), early embryos (e) and somatic cells of the vulva (v). Scale bar, 10 µm. (**B**) Example of a tomographic slice from control RNAi *C. elegans* from the distal region of the gonad. (**C**) Pachytene region of the gonad of a control animal. (**D**) Somatic vulva cell of a control animal. Scale bars, 100 nm. (**E**) Examples of a tomographic slice from *rpoa‐1(RNAi) C. elegans* from the distal region of the gonad. (**F**) Pachytene region of the gonad of a *rpoa‐1(RNAi)* animal. (**G**) Somatic vulva cell of a *rpoa‐1(RNAi)* animal. Scale bars, 100 nm. (**H**) Numbers of ribosomes per cubic micrometre of cytoplasm in the distal region, the pachytene region and in somatic (vulva) cells in both control (black) and *rpoa‐1(RNAi)* worms (orange) for three different voxel count thresholds. The single measurements are shown as dots, the mean is represented by a horizontal line and the whiskers indicate the standard deviation. After performing Welch's two‐sided *t*‐test, the resulting levels of significance in between control and *rpoa‐1(RNAi)* condition are indicated (***p < 0.001; **p < 0.01; *p < 0.05; n.s.: not significant). (**I**) Overview TEM image of a *rpoa‐1(RNAi)* hermaphrodite, showing the vulva (v), the gonad (g) and multiple embryos (e). Mutant worms show a reduced number of embryos compared to wild‐type worms and ‘unoccupied’ space inside the gonad. The gonad itself, although not fully visible in this image, appears morphologically intact. Scale bar, 100 nm.

To apply the developed electron tomography method to another model system, the total number of ribosomes was determined in cells of the nematode *Caenorhabditis elegans* (Figure 4A). For this purpose, semithick sections of high‐pressure frozen samples prepared for electron tomography were used. Specifically, ribosome number was analysed in both germ cells and somatic cells of four RNAi control animals (hermaphrodites). More specifically, a tomogram was acquired from cells in the distal (Figure 4B) and pachytene (middle) regions of the gonad from each individual worm (Figure 4C), and from one somatic cell of the vulva (Figure 4D). Analysis of the distal gonadal region revealed an average total number of ribosomes per µm^3^ of 20,618 ± 3566 (with a voxel count threshold of 25), 15,198 ± 2914 (with a voxel count threshold of 50) and 8626 ± 2202 (with a voxel count threshold of 100) in volumes of 1 µm^3^. In addition, 18,950 ± 5931 ribosomes (with a voxel count threshold of 25), 13,283 ± 4156 ribosomes (with a voxel count threshold of 50) and 6873 ± 1895 ribosomes (with a voxel count threshold of 100) were detected in the pachytene region. Moreover, an average of 11,599 ± 4448 (voxel count threshold of 25), 8958 ± 3077 (voxel count threshold of 50) and 5472 ± 1776 (voxel count threshold of 100) ribosomes per µm^3^ were detected in somatic cells of the vulva (Data S7). These findings revealed that the number of ribosomes per µm^3^ was not uniform across cells of different tissue origin but varied between different cell types. In conclusion, electron tomography is a suitable technique for detecting and comparing ribosome numbers in different tissues within the same organisms.

*C. elegans* is well known for protein‐deletion experiments using RNA‐mediated interference (RNAi).^17^ This method can easily be applied to manipulate the expression level of specific proteins in hermaphrodites. Therefore, the objective was to determine ribosome numbers in electron tomograms of worms with reduced RPOA‐1 expression. RPOA‐1 encodes a DNA‐directed RNA polymerase I subunit involved in the transcription of structural RNAs of the ribosomal subunits.^35^ It was hypothesised that an RNAi‐mediated reduction of this subunit would compromise DNA binding and reduce the DNA‐directed 5'–3' RNA polymerase activity. This protein depletion should, therefore, lead to a statistically significant reduction in ribosome numbers in worm tissues. To this end, *rpoa‐1(RNAi)* worms were prepared for electron tomography by high‐pressure freezing, freeze‐substitution and resin embedding, as performed for control animals. Electron tomograms were then acquired from both germ cells and somatic cells at positions similar to those analysed in control worms. Compared to control animals, a lower total number of ribosomes was apparent in the *rpoa‐1(RNAi)* worms, as confirmed by visual inspection of selected tomographic slices from various cell types (Figure 4E–G). Quantitative analysis revealed that distal cells of the gonad contained, on average, 12,817 ± 4956 (voxel count threshold of 25), 9485 ± 3507 voxel count threshold of 50) and 5312 ± 1859 (voxel count threshold of 100) ribosomes per µm^3^, whereas cells in pachytene showed 12,984 ± 4432 (voxel count threshold of 25), 9029 ± 3855 (voxel count threshold of 50) and 4311 ± 2224 (voxel count threshold of 100) ribosomes per µm^3^. Additionally, somatic cells of the vulva showed 9089 ± 5448 (voxel count threshold of 25), 5508 ± 3294 (voxel count threshold of 50) and 2708 ± 1615 (voxel count threshold of 100) ribosomes per µm^3^. Using Welch's two‐sided *t*‐test, a significantly higher number of ribosomes was detected in the distal part of the gonad and in the pachytene region of control animals compared to similar regions in *rpoa‐1(RNAi)* worms (see Data S8). However, the number of ribosomes in somatic cells of the vulva did not differ between control and *rpoa‐1(RNAi)* worms at the 25 voxel count threshold, while a significant difference was observed at the 50 and 100 voxel count thresholds (Figure 4H and Data S8). Thus, the effect of RPOA‐1 depletion on gonadal versus somatic cells appears to differ. Interestingly, RNAi‐treated worms showed a reduced number of embryos and exhibited large empty spaces inside the gonad (Figure 4I). However, the gonad itself and the ultrastructure of the vulva appeared to be intact.

In conclusion, the developed microscopic approach is not only applicable for a systematic comparison of ribosome numbers in cells of different tissue origins but also for a detailed analysis of control versus RNAi‐treated *C. elegans* hermaphrodites.

## 4 DISCUSSION

Ribosomes can be reliably detected using conventional transmission electron microscopy.^26^, ^35^, ^36^, ^37^, ^38^, ^39^ Here, we present a new method for quantifying ribosome numbers in electron tomograms acquired from specimens after high‐pressure freezing, freeze substitution and resin embedding.

### 4.1 Methodological considerations

For the acquisition of tomograms from hTERT‐RPE‐1 cells, several critical steps in sample preparation were considered. (1) Trypsin treatment during cell harvesting for high‐pressure freezing was avoided, as cells treated with trypsin‐EDTA exhibited ribosome clustering.^40^ Instead, we employed the shake‐off method to detach cells from the support, aiming to minimise artefacts caused by the use of chemicals.^41^, ^42^ (2) Relevant to both hTERT‐RPE‐1 cells and *C. elegans* samples embedded in plastic, the electron beam induces collapse of the sections in the electron microscope during data acquisition.^25^ Importantly, a specific shrinkage factor must be applied for each type of resin used in 3D electron microscopy.^26^, ^43^ A mean shrinkage factor was calculated for Epon/Araldite and applied to the Z‐voxel size to correct for collapse in the Z‐dimension, thus correcting the volumetric measurements. By leveraging a large collection of datasets obtained in the lab, it was possible to calculate a robust mean shrinkage factor for Epon/Araldite (see Data S1). (3) Confocal light microscopy was used to determine both the volume of the hTERT‐RPE‐1 cells and that of their nuclei. The volume of the cytoplasm was obtained by subtracting the nuclear volume from the total volume, minus an additional 10% to account for other organelles in the cytoplasm. However, it is possible that the cytoplasmic volume available for ribosome distribution is actually lower due to the presence of non‐ribosomal compartments, such as Golgi, ER, vesicles, and densely packed cytoskeletal regions, including F‐actin. For simplicity, these additional organelles were estimated to occupy approximately 10% of the cytoplasmic volume. If this estimation is too low, our tomography‐based calculations could overestimate the total number of ribosomes per cell. Additionally, we selected small tomographic subvolumes in areas that did not contain organelles. Therefore, there are several opportunities to fine‐tune our microscopic approach for quantitative analysis of the total number of ribosomes in whole cells.

The fact that the size of the ribosomes we determined was much smaller than expected also raises the question of how much biological samples shrink during sample preparation. However, this might also suggest that ribosome size measurements obtained using other methods (e.g., single‐particle cryo‐EM or cryo‐tomography) may have a systematic measurement error that has not been considered previously. We attempted to account for this observation by empirically investigating which segments, with a certain number of voxels, should be excluded from the analysis. In this way, we aim to exclude smaller, globular stained proteins or unassembled ribosomal subunits from the quantification. In our analysis, globular objects within the range of 25–830 voxels are considered indicative of ribosomes.

In general, a significantly larger number of tomograms should be analysed in the future to obtain more precise information about the total number of ribosomes in different cell types. Nevertheless, our electron tomography data showed consistent ribosome counts across different hTERT‐RPE‐1 cells, confirming the overall reliability of our newly developed approach. Additionally, this newly developed microscopic workflow enabled direct comparisons of ribosome numbers in different cell types within the same model system, as demonstrated for *C. elegans*.

### 4.2 Quantification of ribosomes in hTERT‐RPE‐1 using capillary electrophoresis

Various methods can be applied for ribosome quantification.^8^ One such method is capillary electrophoresis, a routinely applied technique to measure rRNA concentrations.^44^ Capillary electrophoresis is commonly used for RNA analysis due to the migration of the negatively charged RNAs toward the anode in an electric field.^45^ This method, however, has limitations related to the threshold or baseline adjustments of the RNA peaks, as well as RNA extraction and purification steps. In the experiments presented here, the biochemical method yielded approximately the same ribosome count as the electron tomography approach for a voxel count threshold of 25, supporting the reliability of this method for yielding accurate results although we cannot fully explain why the measured ribosome volume differs from the theoretical value. Nevertheless, the fact that the average RNA concentration derived from two million cells was approximately double that from one million cells (see Figure 3C) further supports the consistency of the biochemical measurement. These experiments were performed on cells harvested before the fifth passage to avoid the selection of lab‐derived phenotypes that could potentially affect ribosome numbers, as reported for Caco‐2 cells.^46^ Using capillary electrophoresis, the quality of RNA samples was verified through the RNA integrity number (RIN) and the 18S/28S rRNA ratio. High RIN values (> 9.5) and appropriate rRNA ratios (1.9:1.0 to 2.0:1.0) indicated non‐degraded RNA, ensuring accurate ribosome quantification.^33^, ^47^, ^48^, ^49^ Variations in total RNA concentrations between samples may arise from differences in sample acquisition and RNA isolation.^50^ Despite this, consistent numbers of 18S and 28S particles were observed across different sample concentrations, reflecting the co‐transcription of these subunits.^51^ Overall, the biochemical quantitative analysis of rRNA relies on numerous factors and may consistently underestimate the total ribosome count due to RNA loss at each purification step, with the inherent risk of RNA degradation.

### 4.3 Tomographic quantification of ribosomes in *C. elegans* samples

It has been previously shown that interfering with one component of RNA polymerase I causes a disruption in transcriptional activity, thereby affecting ribosome synthesis.^52^ In more detail, it has been shown that a mutation in the *C. elegans* RPOA‐2 RNA polymerase I B subunit results in cell apoptosis and a decrease in ribosomal protein number to 70% compared to wild‐type levels. It has also been demonstrated that fewer functional ribosomes or a lower number of ribosomes cause cell death.^53^ Taking these previous findings into account, control worms were expected to exhibit a higher number of ribosomes compared to *rpoa‐1(RNAi)* worms in both germ and somatic cells. Indeed, the RNAi‐treated worms showed significantly lower numbers of ribosomes in the two selected areas of the gonad. However, the somatic cells of the vulva appeared to be unaffected in a similar manner, possibly due to their state of differentiation. The gonad is highly active in producing large amounts of RNA^54^ and protein,^55^ both needed to proceed with the meiotic program.^56^ This includes the development and maturation of germ cells,^57^ and the production of oocytes that are capable of undergoing the first rounds of mitotic divisions due to maternal loading.^58^, ^59^ As a reproductive organ, the gonad may therefore be more prone to regulation of ribosome number and/or more sensitive to RNA‐mediated interference in general compared to the somatic cells of the vulva. A lower frequency of cell division rounds or a reduced level of protein synthesis in somatic cells could potentially lead to a reduction in ribosomal particles compared to gonadal cells. This possible correlation, however, needs to be analysed in more detail by applying both the presented microscopic method as well as the biochemical approaches.

Taken together, electron tomography can be used for direct quantitative analysis of ribosome numbers in different cell types and in the context of complex tissues. A combination of 3D electron microscopy and biochemistry will open up new possibilities for a comparative and systematic analysis of ribosome distribution in cells or tissues from different model systems in addition to the established methods.

## Supporting information



Supporting Information

Supporting Information

Supporting Information

Supporting Information

Supporting Information

Supporting Information

Supporting Information

Supporting Information

Supporting Information

Supporting Information

Supporting Information

Supporting Information
