# Supplementary figures and images for: In situ quantification of ribosome number by electron tomography
Source: J Microsc. 2025 Jan 15;299(3):212–27. doi: 10.1111/jmi.13380 (PMC12352020; doi:10.1111/jmi.13380)

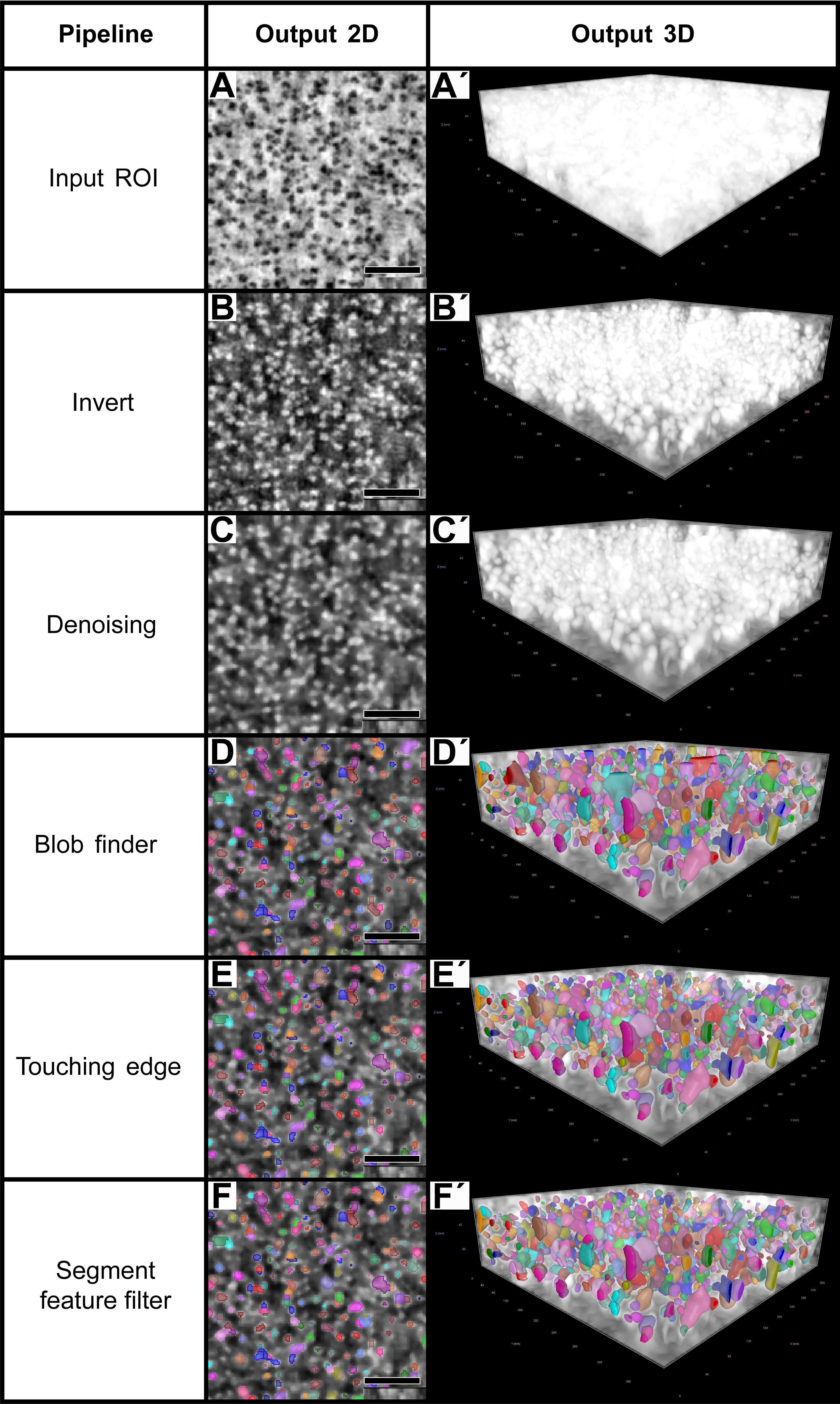

Supplement: Supplementary file 1 — Supporting Information [file JMI-299-212-s001.tif]

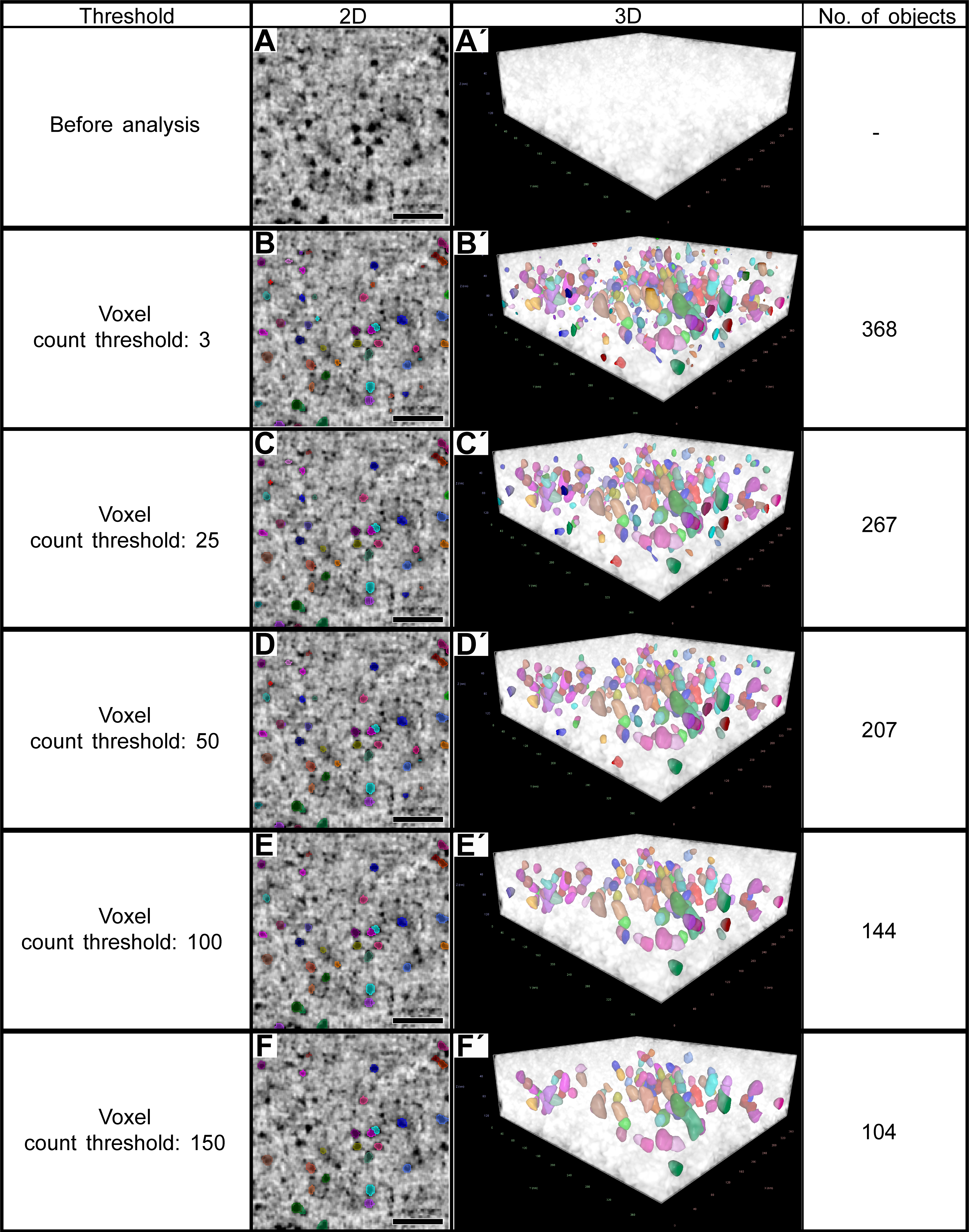

Supplement: Supplementary file 2 — Supporting Information [file JMI-299-212-s009.tif]

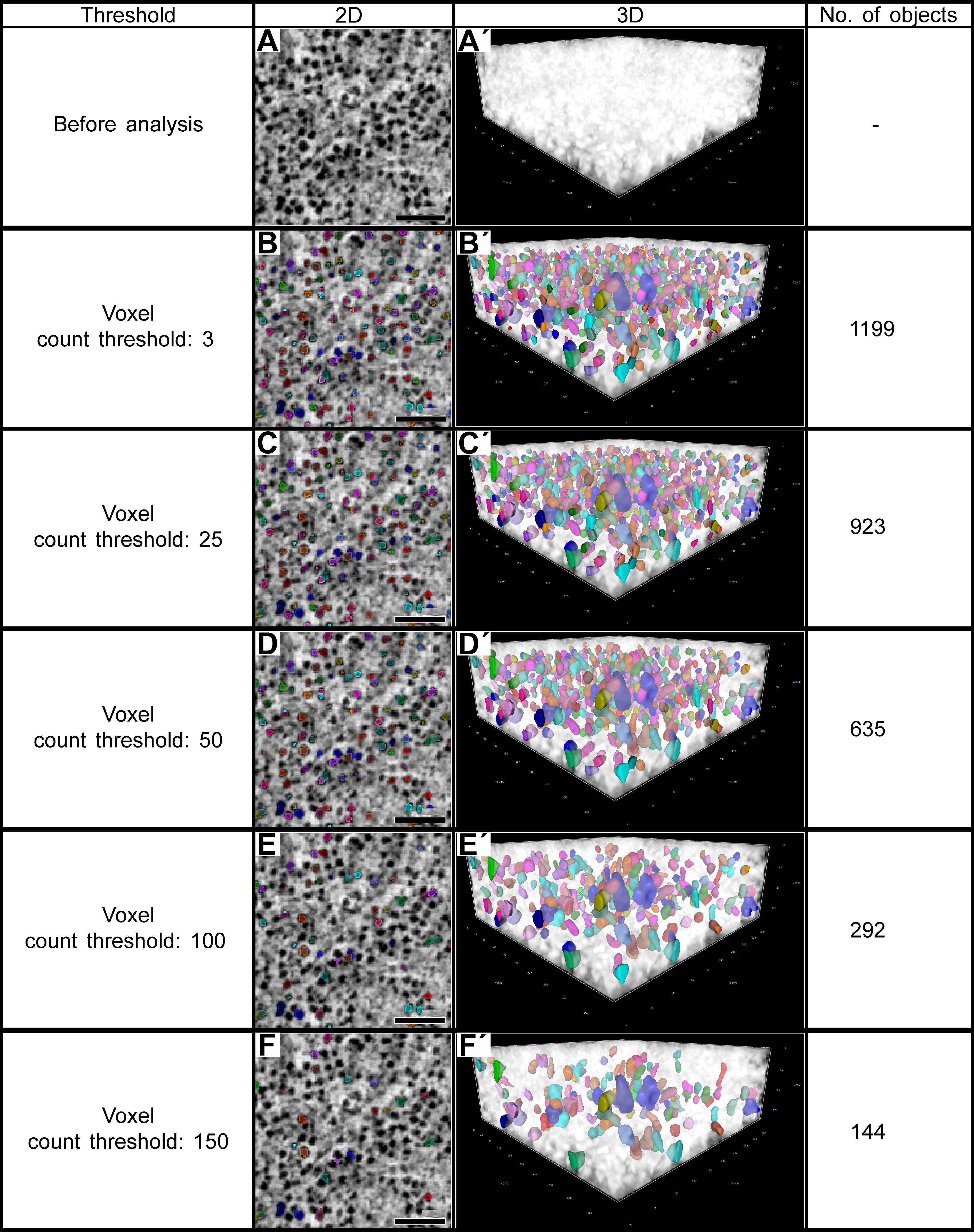

Supplement: Supplementary file 3 — Supporting Information [file JMI-299-212-s003.tif]
