# Supplementary material for: In situ quantification of ribosome number by electron tomography
Source: J Microsc. 2025 Jan 15;299(3):212–27. doi: 10.1111/jmi.13380 (PMC12352020; doi:10.1111/jmi.13380)
Supplement: Supplementary file 4 — Supporting Information [file JMI-299-212-s004.docx]

**Legends for Suppl. Figures**

**Suppl. Fig. 1. Image processing workflow.** (**A**) Cropped-out region of interest (ROI) from an electron tomogram is loaded into Arivis Vision4D. (**B**) The whole data set is then inverted. (**C**) For denoising, the median filter algorithm is used with a diameter of 3 pixels (7.72 nm). (**D**) The tool ‘Blob Finder’ is applied. (**E**) An edge touching filter is used to remove all segments which touched the first and last Z-plane as these planes are always blurry due to the physical section border and to exclude potential colloidal gold fiducial markers. (**F**) As a final step, all segments with a voxel count smaller than a fixed threshold are filtered out. Thresholds of 25, 50, and 100 voxels are chosen.

**Suppl. Fig. 2. Impact of the voxel count threshold on ribosome segmentation in hTERT-RPE-1 cells.** (**A**) Unsegmented ROI cropped from a reconstructed electron tomogram of a hTERT-RPE-1 cell. (**B**) A voxel count threshold of 3 voxels resulted in 368 objects. (**C**) A voxel count threshold of 25 voxels resulted in 267 objects. (**D**) A voxel count threshold of 50 voxels resulted in 207 objects. (**E**) A voxel count threshold of 100 voxels resulted in 144 objects. (**F**) A voxel count threshold of 150 voxels resulted in 104 objects. 2D scale bars, 100 nm.

**Suppl. Fig. 3. Impact of the voxel count threshold on ribosome segmentation in the *C. elegans* gonad.** (**A**) Unsegmented ROI cropped from a reconstructed electron tomogram from the *C. elegans* distal gonad. (**B**) A voxel count threshold of 3 voxels resulted in 1199 objects. (**C**) A voxel count threshold of 25 voxels resulted in 923 objects. (**D**) A voxel count threshold of 50 voxels resulted in 635 objects. (**E**) A voxel count threshold of 100 voxels resulted in 292 objects. (**F**) A voxel count threshold of 150 voxels resulted in 144 objects. 2D scale bars, 100 nm.
