# Supplementary material for: In situ quantification of ribosome number by electron tomography
Source: J Microsc. 2025 Jan 15;299(3):212–27. doi: 10.1111/jmi.13380 (PMC12352020; doi:10.1111/jmi.13380)
Supplement: Supplementary file 6 — Supporting Information [file JMI-299-212-s010.pdf]

Assay Class: Eukaryote Total RNA Nano
Data Path: C:\...Eukaryote Total RNA Nano_DEDAE01485_2022-06-14_15-36-23.xad

Created: 6/14/2022 3:36:23 PM
Modified: 6/14/2022 4:08:38 PM

Electrophoresis File Run Summary

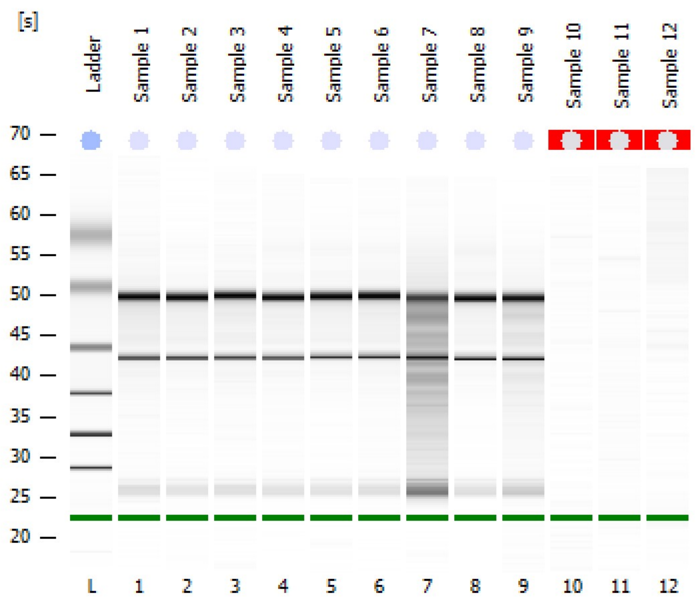

Instrument Information:

Instrument Name: DEDAE01485 Firmware: C.01.069

Serial#: DEDAE01485 Type: G2939B

Assay Information:

Assay Origin Path: C:\Program Files (x86)\Agilent\2100 bioanalyzer\2100 expert\assays\RNA\Eukaryote Total RNA Nano Series II.xsy

Assay Class: Eukaryote Total RNA Nano

Version: 2.6

Assay Comments: Total RNA Analysis ng sensitivity (Eukaryote)

© Copyright 2003 - 2009 Agilent Technologies, Inc.

Chip Information:

Chip Lot #:

Reagent Kit Lot #:

Chip Comments:

Sample 1

RIN: 9.50

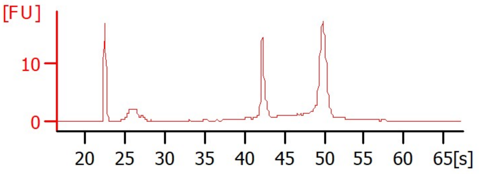

Sample 2

RIN: 9.80

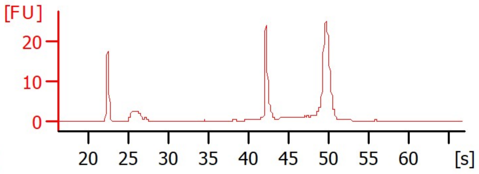

Sample 3

RIN: 9.80

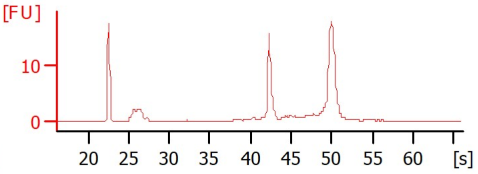

Sample 4

RIN: 9.90

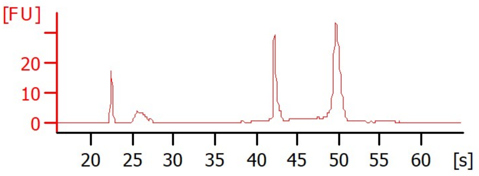

Sample 5

RIN:10

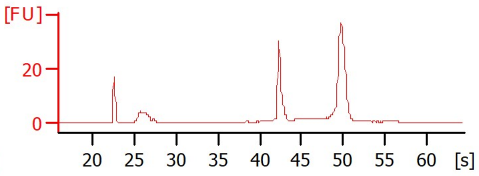

Sample 6

RIN: 9.80

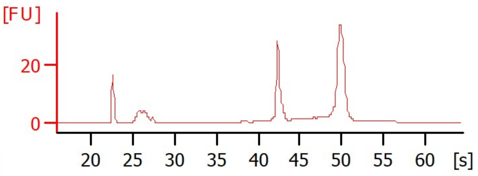

Sample 7

RIN: 5.80

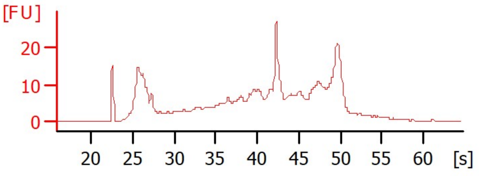

Sample 8

RIN: 9.70

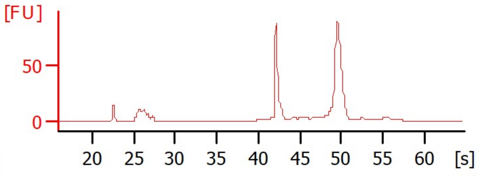

Sample 9

RIN: 8.40

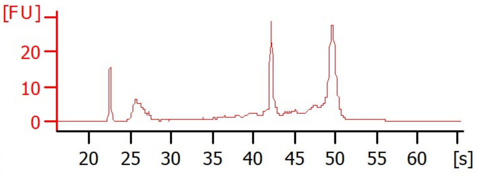

Sample 10

RIN N/A

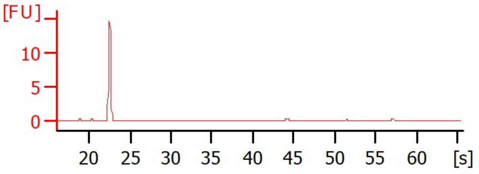

Sample 11

RIN N/A

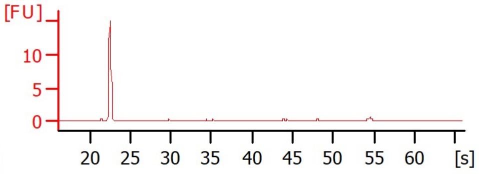

Sample 12

RIN N/A

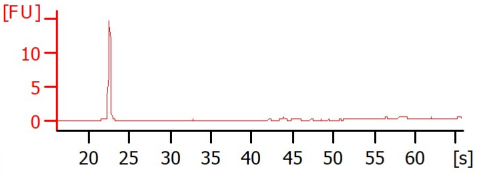

Assay Class: Eukaryote Total RNA Nano
Data Path: C:\...Eukaryote Total RNA Nano_DEDAE01485_2022-06-14_15-36-23.xad
Created: 6/14/2022 3:36:23 PM
Modified: 6/14/2022 4:08:38 PM

Electrophoresis File Run Summary (Chip Summary)

Sample Name	Sample Comment	Status	Result Label	Result Color
Sample 1		✔	RIN: 9.50	
Sample 2		✔	RIN: 9.80	
Sample 3		✔	RIN: 9.80	
Sample 4		✔	RIN: 9.90	
Sample 5		✔	RIN:10	
Sample 6		✔	RIN: 9.80	
Sample 7		✔	RIN: 5.80	
Sample 8		✔	RIN: 9.70	
Sample 9		✔	RIN: 8.40	
Sample 10		✔	RIN N/A	
Sample 11		✔	RIN N/A	
Sample 12		✔	RIN N/A	
Ladder		✔	All Other Samples	

Chip Lot # **Reagent Kit Lot #**

Chip Comments :

© Copyright 2003 - 2018 Agilent, Inc.

Assay Class: Eukaryote Total RNA Nano
Data Path: C:\...Eukaryote Total RNA Nano_DEDAE01485_2022-06-14_15-36-23.xad
Created: 6/14/2022 3:36:23 PM
Modified: 6/14/2022 4:08:38 PM

Electrophoresis Assay Details

General Analysis Settings

Number of Available Sample and Ladder Wells (Max.) : 13
Minimum Visible Range [s] : 17
Maximum Visible Range [s] : 70
Start Analysis Time Range [s] : 19
End Analysis Time Range [s] : 69
Ladder Concentration [ng/µl] : 150
Lower Marker Concentration [ng/µl] : 0
Upper Marker Concentration [ng/µl] : 0
Used Lower Marker for Quantitation
Standard Curve Fit is Logarithmic
Show Data Aligned to Lower Marker

Integrator Settings

Integration Start Time [s] : 19
Integration End Time [s] : 69
Slope Threshold : 0.6
Height Threshold [FU] : 0.5
Area Threshold : 0.2
Width Threshold [s] : 0.5
Baseline Plateau [s] : 6

Filter Settings

Filter Width [s] : 0.5
Polynomial Order : 4

Ladder

Ladder Peak	Size
1	25
2	200
3	500
4	1000
5	2000
6	4000

 © Copyright 2003 - 2018 Agilent, Inc.

Assay Class: Eukaryote Total RNA Nano
Data Path: C:\...Eukaryote Total RNA Nano_DEDAE01485_2022-06-14_15-36-23.xad

Created: 6/14/2022 3:36:23 PM
Modified: 6/14/2022 4:08:38 PM

Electropherogram Summary

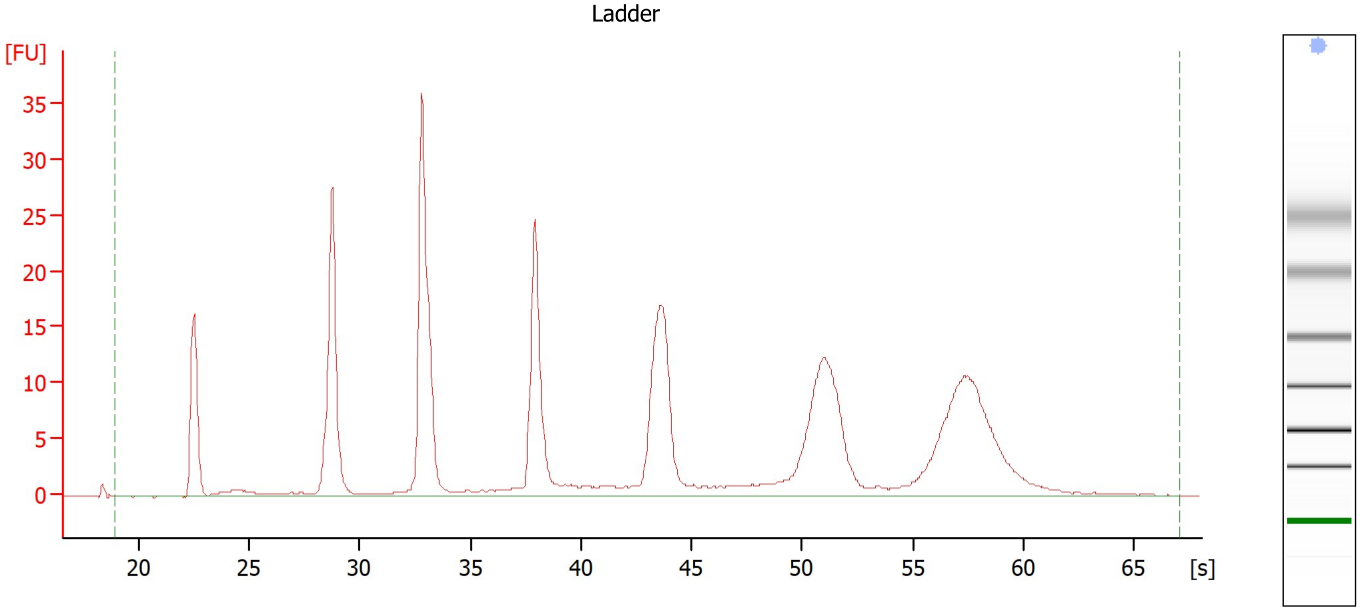

Overall Results for Ladder

RNA Area:	291.0	Result Flagging Color:	
RNA Concentration:	150 ng/µl	Result Flagging Label:	All Other Samples

© Copyright 2003 - 2018 Agilent, Inc.

Assay Class:	Eukaryote Total RNA Nano	Created:	6/14/2022 3:36:23 PM
Data Path:	C:\...Eukaryote Total RNA Nano_DEDAE01485_2022-06-14_15-36-23.xad	Modified:	6/14/2022 4:08:38 PM

Electropherogram Summary Continued ...

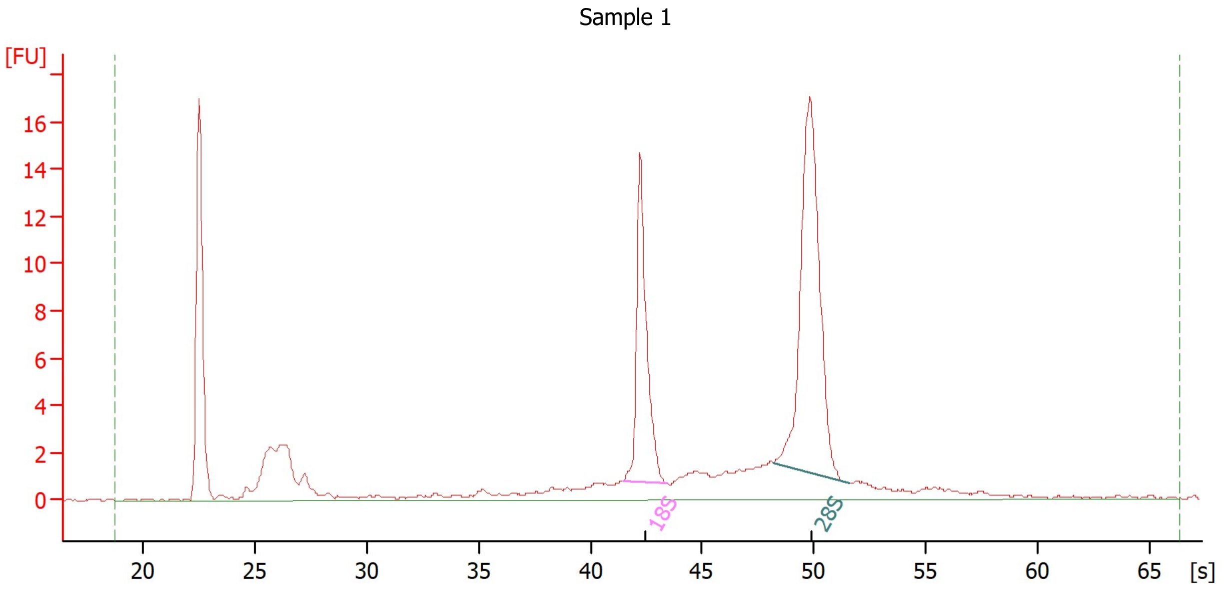

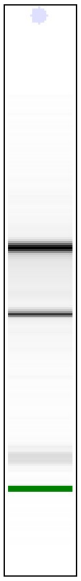

Overall Results for sample 1 : Sample 1

RNA Area:	102.8	RNA Integrity Number (RIN):	9.5 (B.02.10)
RNA Concentration:	53 ng/µl	Result Flagging Color:	
rRNA Ratio [28s / 18s]:	1.8	Result Flagging Label:	RIN: 9.50

Fragment table for sample 1 : Sample 1

Name	Start Time [s]	End Time [s]	Area	% of total Area
18S	41.49	43.48	16.9	16.5
28S	48.17	51.59	31.1	30.2

 © Copyright 2003 - 2018 Agilent, Inc.

Assay Class: Eukaryote Total RNA Nano
Data Path: C:\...Eukaryote Total RNA Nano_DEDAE01485_2022-06-14_15-36-23.xad

Created: 6/14/2022 3:36:23 PM
Modified: 6/14/2022 4:08:38 PM

Electropherogram Summary Continued ...

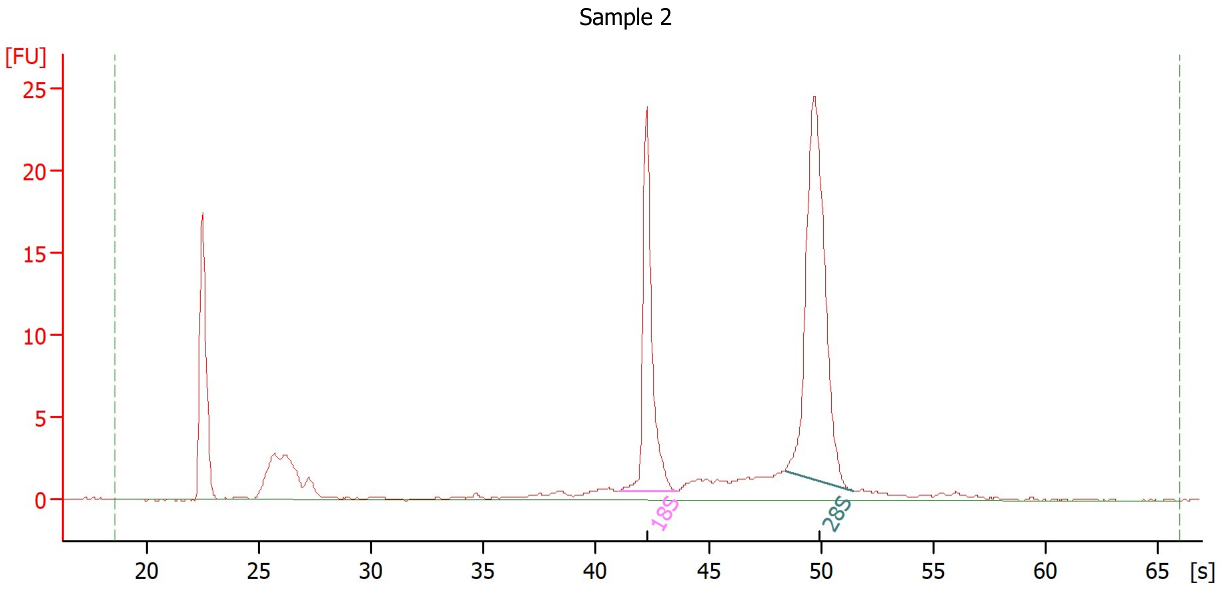

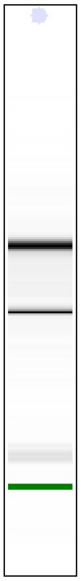

Overall Results for sample 2 : Sample 2

RNA Area:	122.3	RNA Integrity Number (RIN):	9.8 (B.02.10)
RNA Concentration:	63 ng/µl	Result Flagging Color:	
rRNA Ratio [28s / 18s]:	1.9	Result Flagging Label:	RIN: 9.80

Fragment table for sample 2 : Sample 2

Name	Start Time [s]	End Time [s]	Area	% of total Area
18S	40.99	43.61	24.3	19.8
28S	48.39	51.45	45.2	37.0

 © Copyright 2003 - 2018 Agilent, Inc.

Assay Class: Eukaryote Total RNA Nano
Data Path: C:\...Eukaryote Total RNA Nano_DEDAE01485_2022-06-14_15-36-23.xad

Created: 6/14/2022 3:36:23 PM
Modified: 6/14/2022 4:08:38 PM

Electropherogram Summary Continued ...

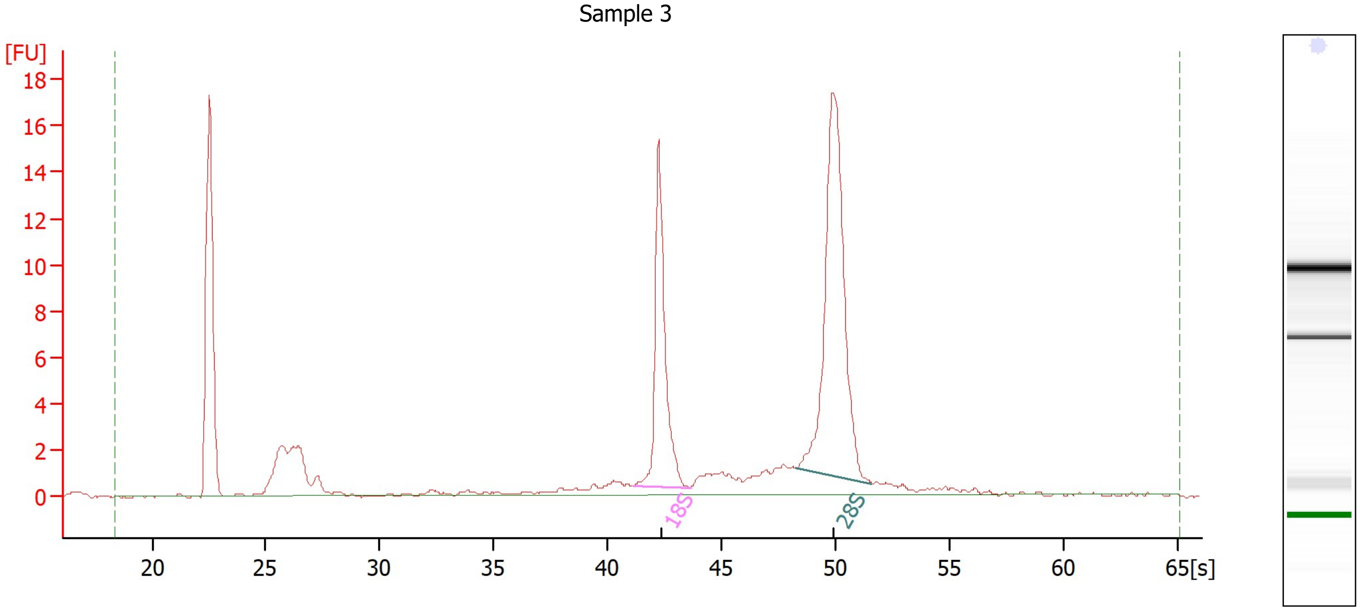

Overall Results for sample 3 : Sample 3

RNA Area:	90.7	RNA Integrity Number (RIN):	9.8 (B.02.10)
RNA Concentration:	47 ng/µl	Result Flagging Color:	
rRNA Ratio [28s / 18s]:	1.9	Result Flagging Label:	RIN: 9.80

Fragment table for sample 3 : Sample 3

Name	Start Time [s]	End Time [s]	Area	% of total Area
18S	41.08	43.63	16.9	18.6
28S	48.25	51.60	31.8	35.1

 © Copyright 2003 - 2018 Agilent, Inc.

Assay Class:	Eukaryote Total RNA Nano	Created:	6/14/2022 3:36:23 PM
Data Path:	C:\...Eukaryote Total RNA Nano_DEDAE01485_2022-06-14_15-36-23.xad	Modified:	6/14/2022 4:08:38 PM

Electropherogram Summary Continued ...

Sample 4

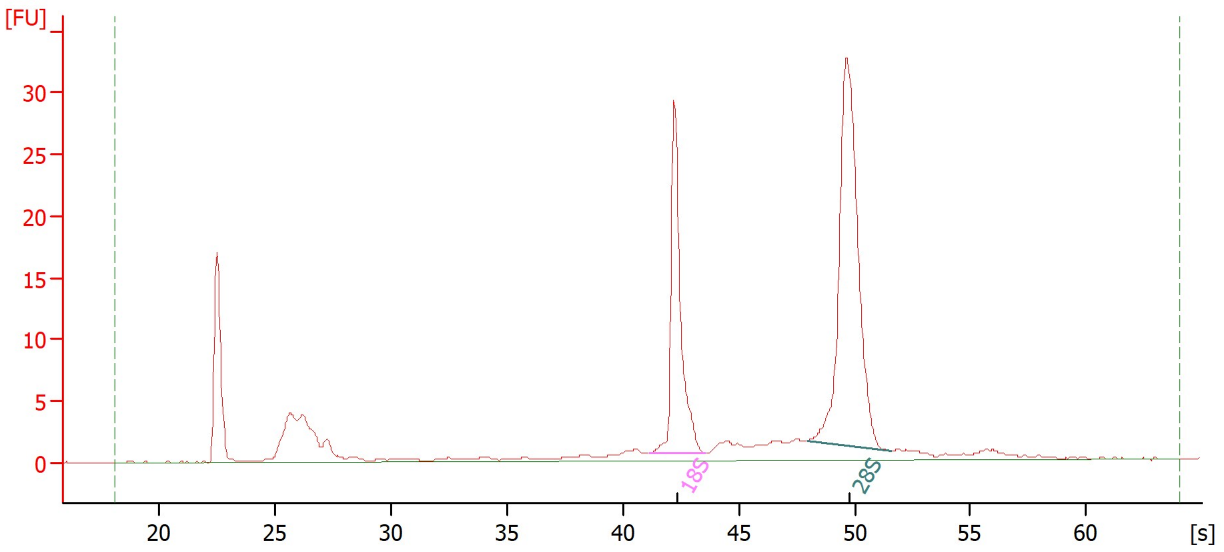

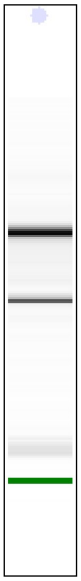

Overall Results for sample 4 : Sample 4

RNA Area:	157.3	RNA Integrity Number (RIN):	9.9 (B.02.10)
RNA Concentration:	81 ng/µl	Result Flagging Color:	
rRNA Ratio [28s / 18s]:	1.9	Result Flagging Label:	RIN: 9.90

Fragment table for sample 4 : Sample 4

Name	Start Time [s]	End Time [s]	Area	% of total Area
18S	41.10	43.52	31.0	19.7
28S	47.97	51.59	59.9	38.0

 © Copyright 2003 - 2018 Agilent, Inc.

Assay Class:	Eukaryote Total RNA Nano	Created:	6/14/2022 3:36:23 PM
Data Path:	C:\...Eukaryote Total RNA Nano_DEDAE01485_2022-06-14_15-36-23.xad	Modified:	6/14/2022 4:08:38 PM

Electropherogram Summary Continued ...

Sample 5

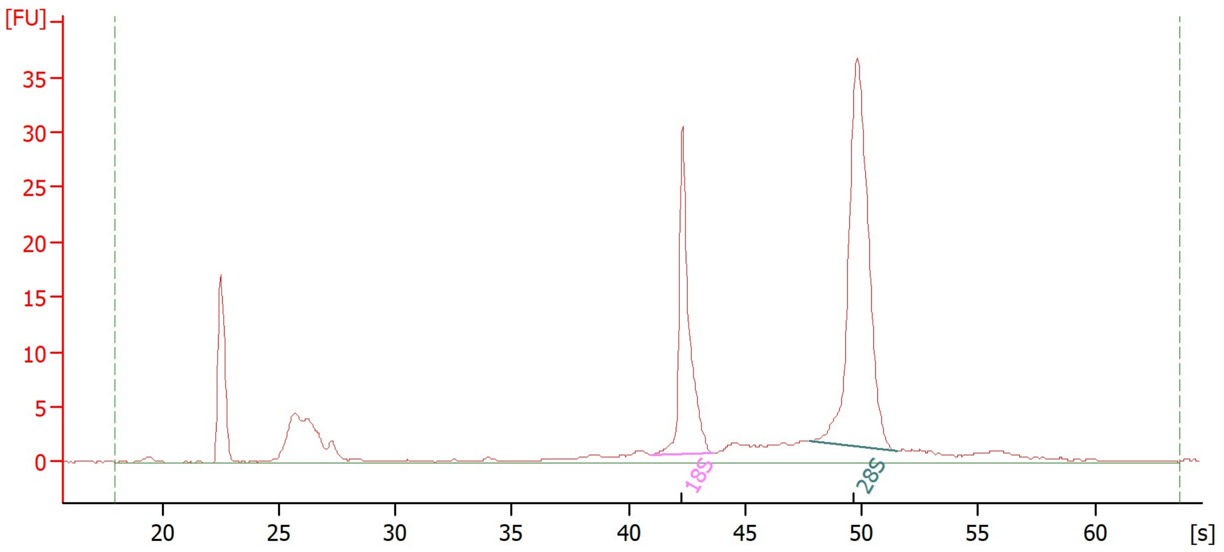

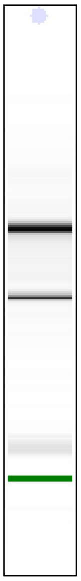

Overall Results for sample 5 : Sample 5

RNA Area:	182.1	RNA Integrity Number (RIN):	10 (B.02.10)
RNA Concentration:	94 ng/µl	Result Flagging Color:	
rRNA Ratio [28s / 18s]:	2.0	Result Flagging Label:	RIN:10

Fragment table for sample 5 : Sample 5

Name	Start Time [s]	End Time [s]	Area	% of total Area
18S	40.99	43.66	34.9	19.1
28S	47.79	51.55	69.6	38.2

 © Copyright 2003 - 2018 Agilent, Inc.

Electropherogram Summary Continued ...

Sample 6

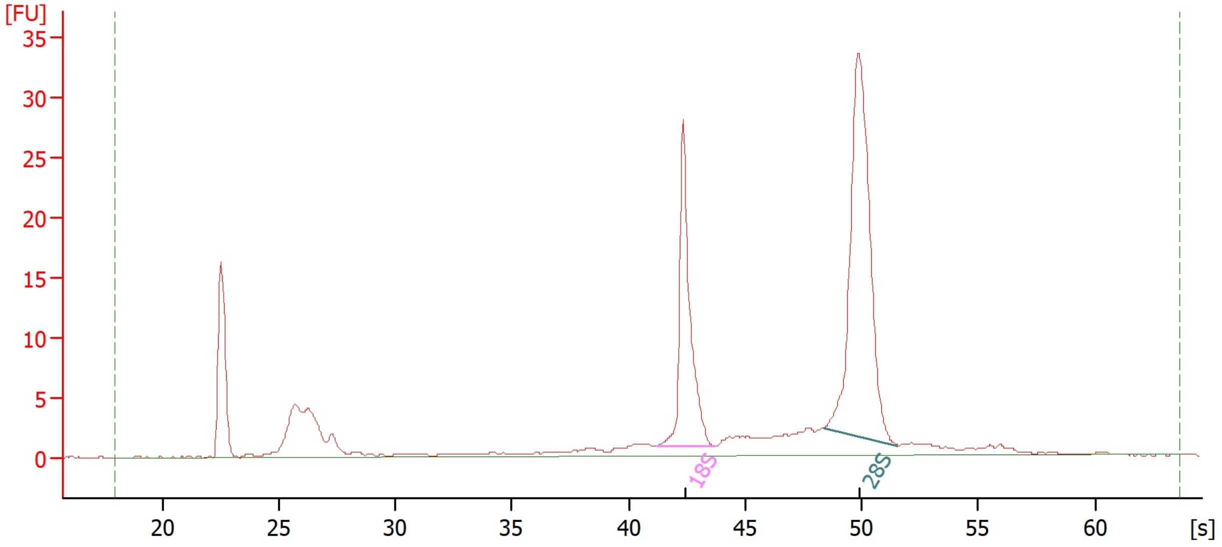

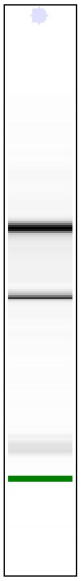

Overall Results for sample 6 : **Sample 6**

RNA Area:	177.7	RNA Integrity Number (RIN):	9.8 (B.02.10)
RNA Concentration:	92 ng/µl	Result Flagging Color:	
rRNA Ratio [28s / 18s]:	1.9	Result Flagging Label:	RIN: 9.80

Fragment table for sample 6 : **Sample 6**

Name	Start Time [s]	End Time [s]	Area	% of total Area
18S	41.22	43.76	32.6	18.4
28S	48.37	51.55	62.3	35.0

 © Copyright 2003 - 2018 Agilent, Inc.

Assay Class:	Eukaryote Total RNA Nano	Created:	6/14/2022 3:36:23 PM
Data Path:	C:\...Eukaryote Total RNA Nano_DEDAE01485_2022-06-14_15-36-23.xad	Modified:	6/14/2022 4:08:38 PM

Electropherogram Summary Continued ...

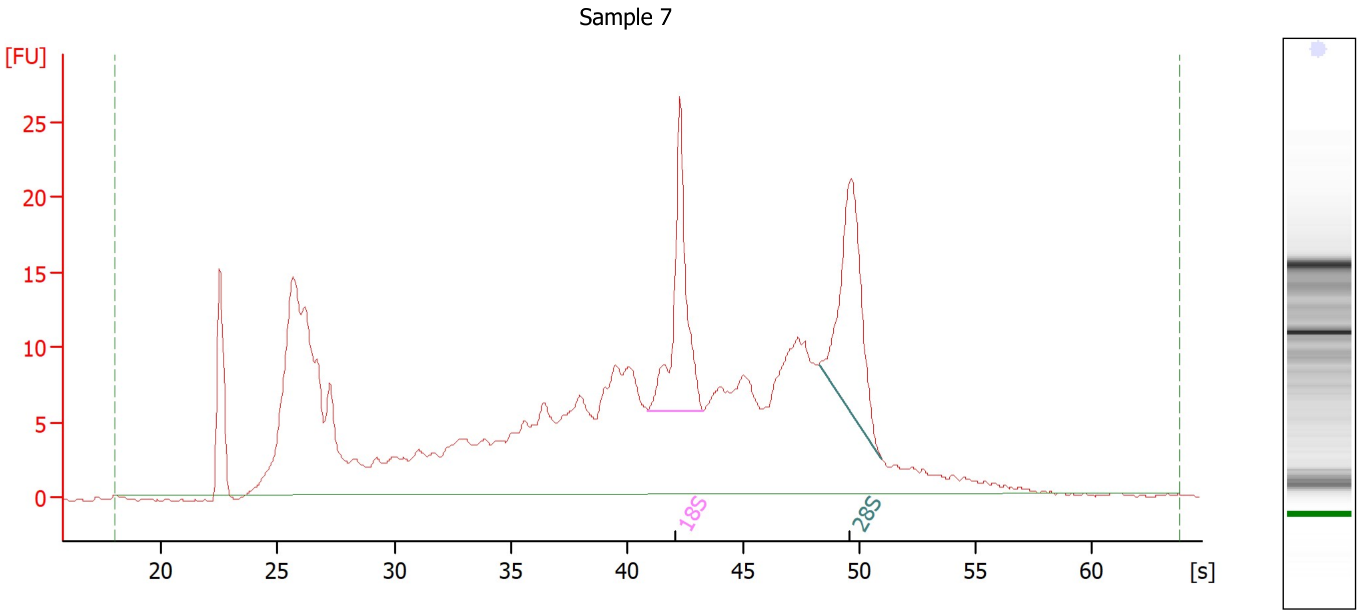

Overall Results for sample 7 : Sample 7

RNA Area:	476.2	RNA Integrity Number (RIN):	5.8 (B.02.10)
RNA Concentration:	245 ng/µl	Result Flagging Color:	
rRNA Ratio [28s / 18s]:	1.1	Result Flagging Label:	RIN: 5.80

Fragment table for sample 7 : Sample 7

Name	Start Time [s]	End Time [s]	Area	% of total Area
18S	40.89	43.29	28.9	6.1
28S	48.28	50.96	32.8	6.9

 © Copyright 2003 - 2018 Agilent, Inc.

Assay Class: Eukaryote Total RNA Nano
Data Path: C:\...Eukaryote Total RNA Nano_DEDAE01485_2022-06-14_15-36-23.xad

Created: 6/14/2022 3:36:23 PM
Modified: 6/14/2022 4:08:38 PM

Electropherogram Summary Continued ...

Sample 8

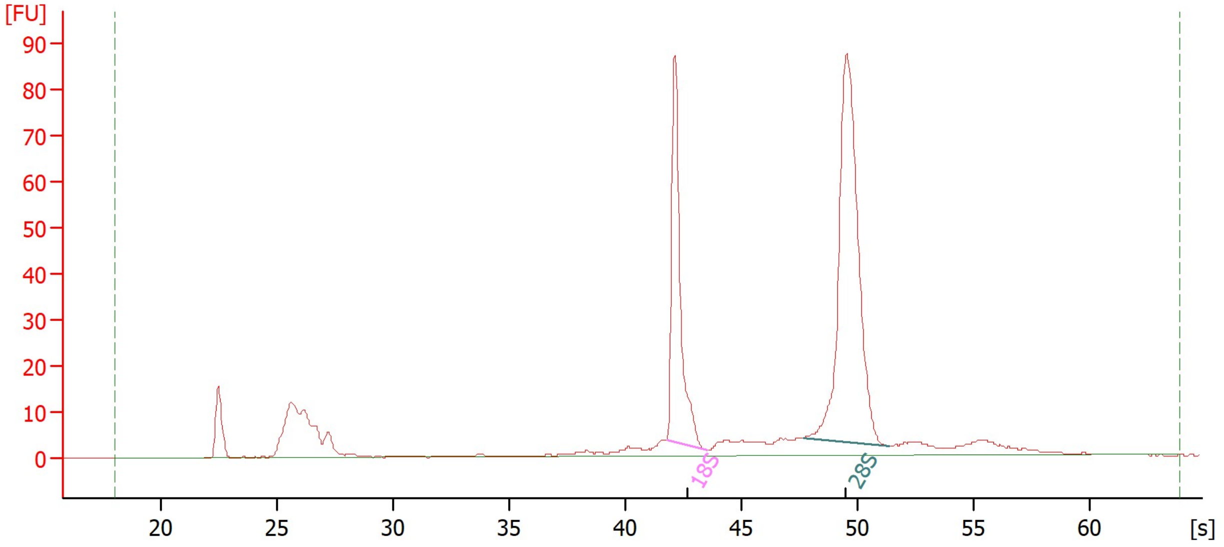

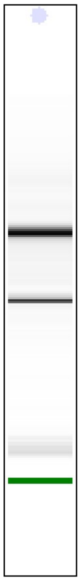

Overall Results for sample 8 : **Sample 8**

RNA Area:	429.3	RNA Integrity Number (RIN):	9.7 (B.02.10)
RNA Concentration:	221 ng/µl	Result Flagging Color:	
rRNA Ratio [28s / 18s]:	2.0	Result Flagging Label:	RIN: 9.70

Fragment table for sample 8 : **Sample 8**

Name	Start Time [s]	End Time [s]	Area	% of total Area
18S	41.81	43.52	82.4	19.2
28S	47.69	51.44	164.1	38.2

© Copyright 2003 - 2018 Agilent, Inc.

Assay Class:	Eukaryote Total RNA Nano	Created:	6/14/2022 3:36:23 PM
Data Path:	C:\...Eukaryote Total RNA Nano_DEDAE01485_2022-06-14_15-36-23.xad	Modified:	6/14/2022 4:08:38 PM

Electropherogram Summary Continued ...

Sample 9

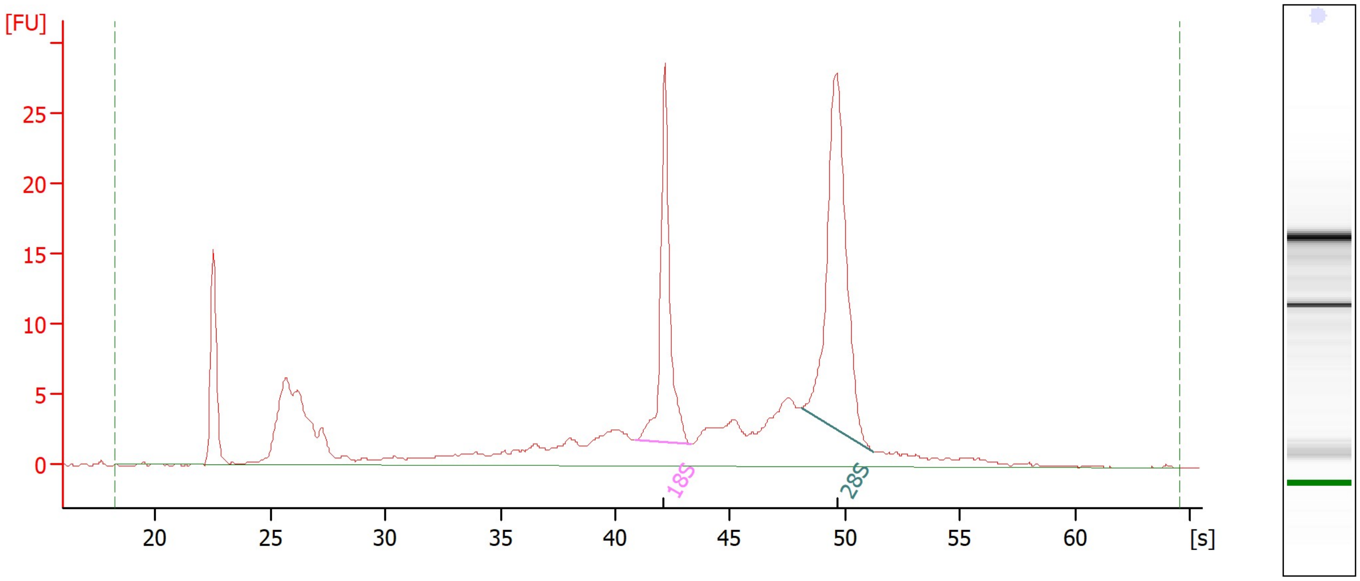

Overall Results for sample 9 : **Sample 9**

RNA Area:	219.8	RNA Integrity Number (RIN):	8.4 (B.02.10)
RNA Concentration:	113 ng/µl	Result Flagging Color:	
rRNA Ratio [28s / 18s]:	1.7	Result Flagging Label:	RIN: 8.40

Fragment table for sample 9 : **Sample 9**

Name	Start Time [s]	End Time [s]	Area	% of total Area
18S	40.88	43.32	29.0	13.2
28S	48.09	51.27	49.4	22.5

 © Copyright 2003 - 2018 Agilent, Inc.

Assay Class:	Eukaryote Total RNA Nano	Created:	6/14/2022 3:36:23 PM
Data Path:	C:\...Eukaryote Total RNA Nano_DEDAE01485_2022-06-14_15-36-23.xad	Modified:	6/14/2022 4:08:38 PM

Electropherogram Summary Continued ...

Sample 10

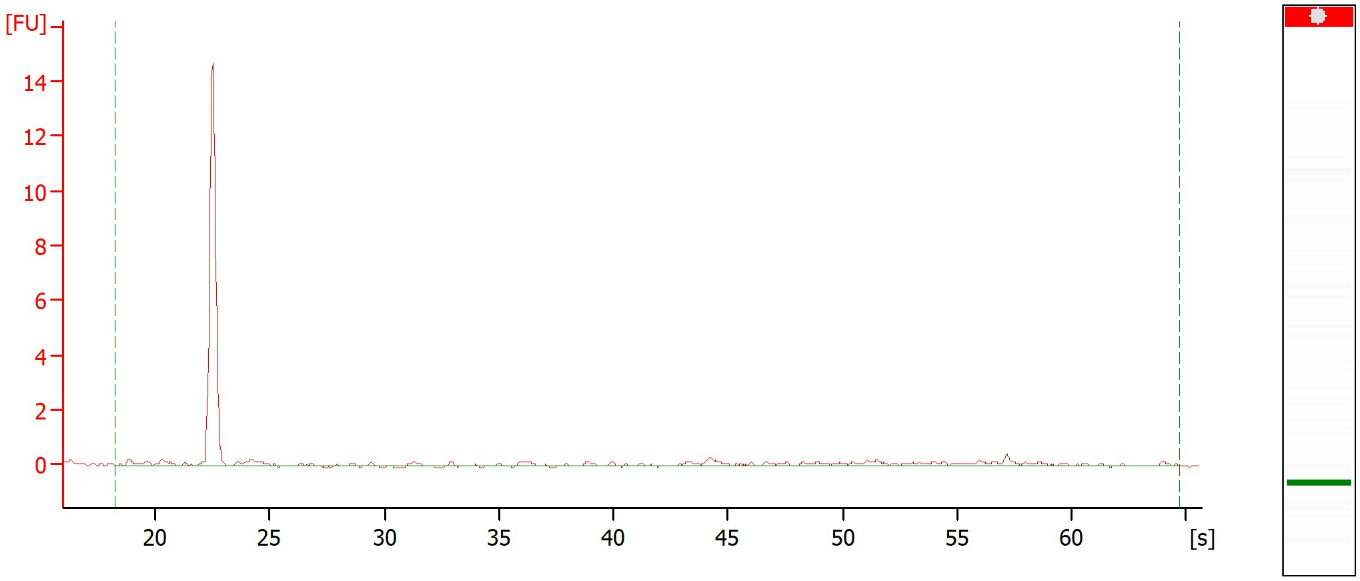

Overall Results for sample 10 : **Sample 10**

RNA Area:	4.6	RNA Integrity Number (RIN):	N/A (B.02.10)
RNA Concentration:	2 ng/µl	Result Flagging Color:	
rRNA Ratio [28s / 18s]:	0.0	Result Flagging Label:	RIN N/A

 © Copyright 2003 - 2018 Agilent, Inc.

Assay Class: Eukaryote Total RNA Nano | Created: 6/14/2022 3:36:23 PM
Data Path: C:\...Eukaryote Total RNA Nano_DEDAE01485_2022-06-14_15-36-23.xad | Modified: 6/14/2022 4:08:38 PM

Electropherogram Summary Continued ...

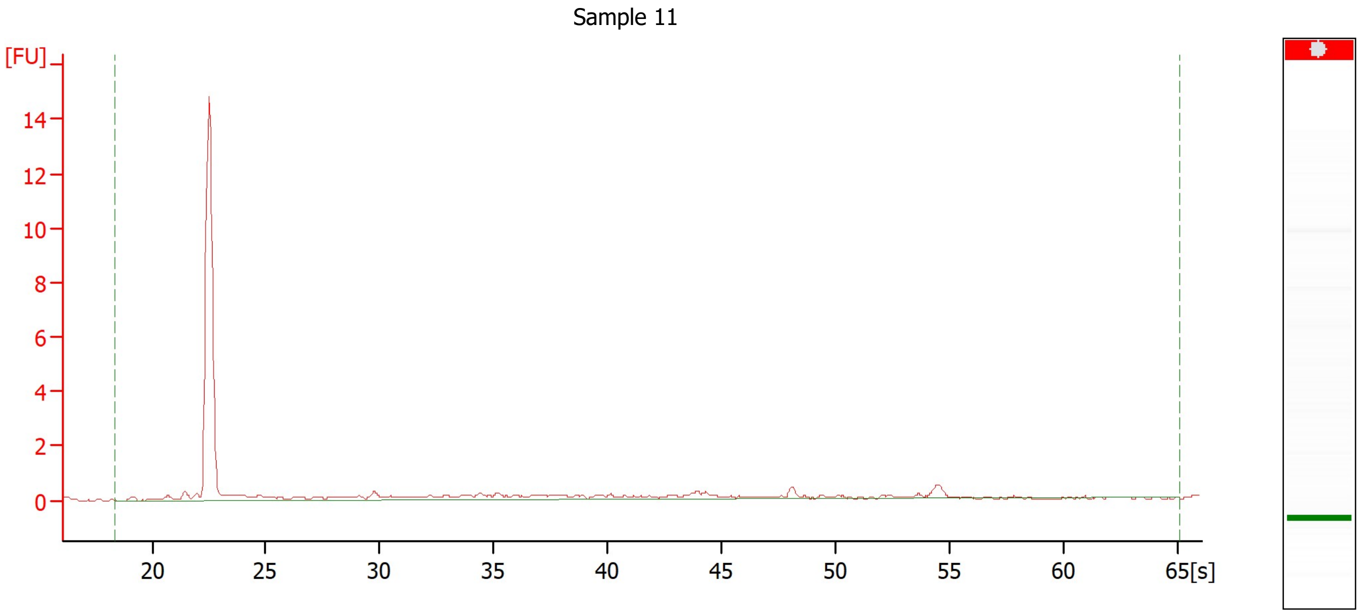

Overall Results for sample 11 : **Sample 11**

RNA Area:	9.8	RNA Integrity Number (RIN):	N/A (B.02.10)
RNA Concentration:	5 ng/µl	Result Flagging Color:	
rRNA Ratio [28s / 18s]:	0.0	Result Flagging Label:	RIN N/A

 © Copyright 2003 - 2018 Agilent, Inc.

Assay Class:	Eukaryote Total RNA Nano	Created:	6/14/2022 3:36:23 PM
Data Path:	C:\...Eukaryote Total RNA Nano_DEDAE01485_2022-06-14_15-36-23.xad	Modified:	6/14/2022 4:08:38 PM

Electropherogram Summary Continued ...

Sample 12

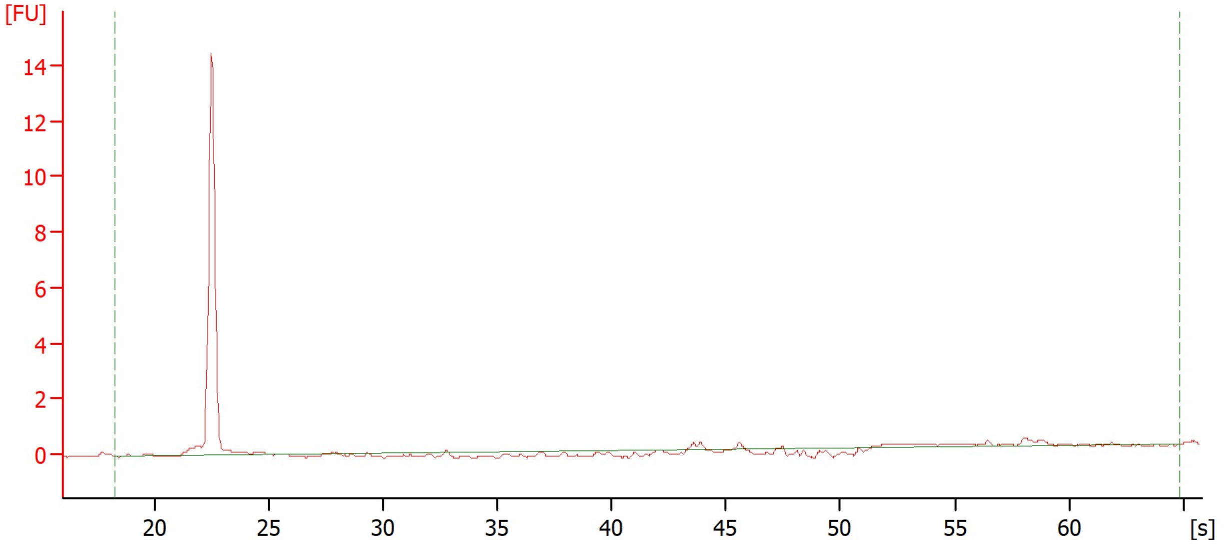

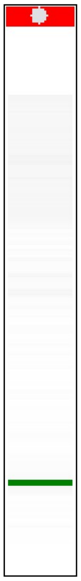

Overall Results for sample 12 : **Sample 12**

RNA Area:	3.2	RNA Integrity Number (RIN):	N/A (B.02.10)
RNA Concentration:	2 ng/µl	Result Flagging Color:	
rRNA Ratio [28s / 18s]:	0.0	Result Flagging Label:	RIN N/A

 © Copyright 2003 - 2018 Agilent, Inc.

Assay Class:	Eukaryote Total RNA Nano	Created:	6/14/2022 3:36:23 PM
Data Path:	C:\...Eukaryote Total RNA Nano_DEDAE01485_2022-06-14_15-36-23.xad	Modified:	6/14/2022 4:08:38 PM

Gel Image

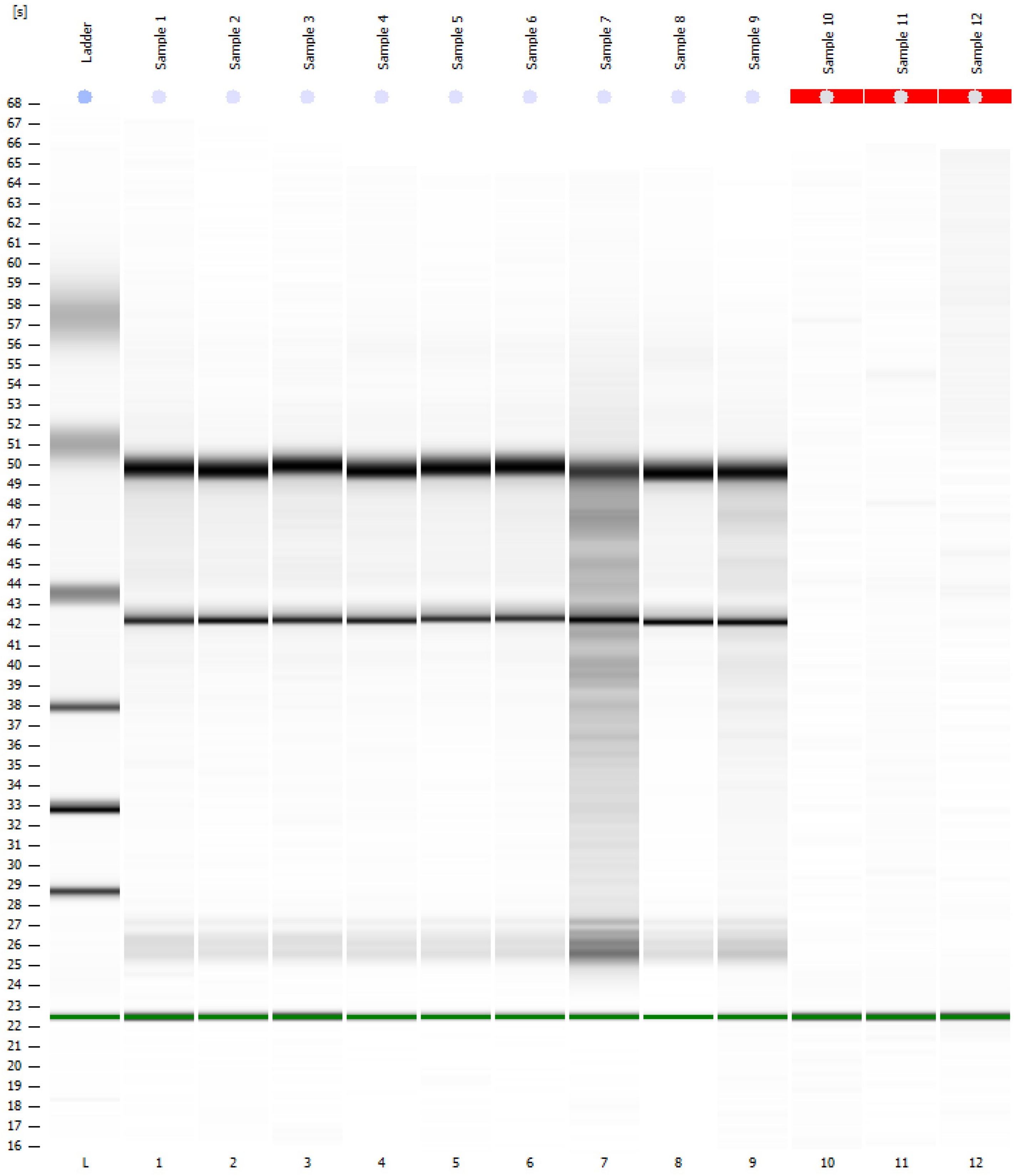

 © Copyright 2003 - 2018 Agilent, Inc.

Assay Class: Eukaryote Total RNA Nano
Data Path: C:\...Eukaryote Total RNA Nano_DEDAE01485_2022-06-14_15-36-23.xad
Created: 6/14/2022 3:36:23 PM
Modified: 6/14/2022 4:08:38 PM

Curves

Standard Curve

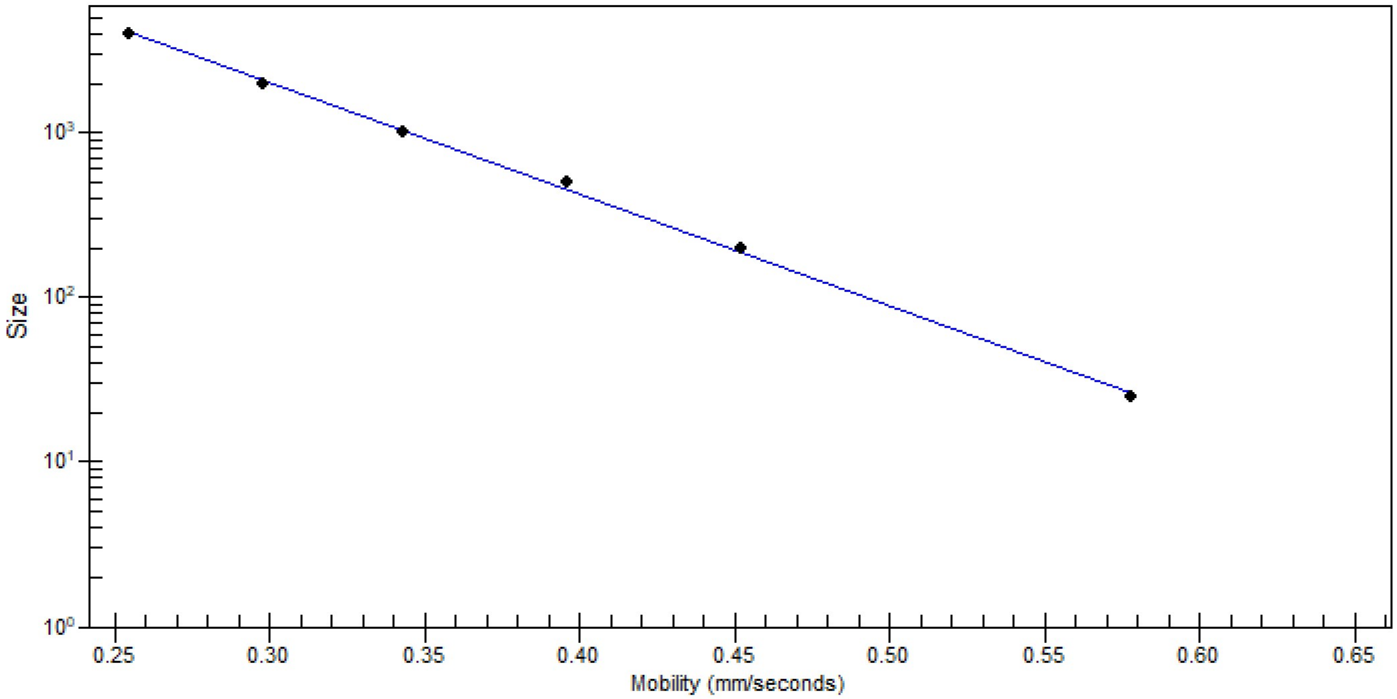

 © Copyright 2003 - 2018 Agilent, Inc.

Assay Class: Eukaryote Total RNA Nano
Data Path: C:\...Eukaryote Total RNA Nano_DEDAE01485_2022-06-14_15-36-23.xad
Created: 6/14/2022 3:36:23 PM
Modified: 6/14/2022 4:08:38 PM

Run Logbook

Description	Number	Source	Category	Sub Category	Time	Time Zone	User	Host
Run ended on port 1 (Number of wells acquired: 13)		Instrument	Run		6/14/2022 4:00:13 PM	(GMT +02:00) W. Europe Standard Time	user_agilent	FMP1
Run started on port 1 (File: C:\Program Files (x86)\Agilent\2100 bioanalyzer\2100 expert\Data\2022-06-14\2100 expert_Eukaryote Total RNA Nano_DEDAE01485_2022-06-14_15-36-23.xad)		Instrument	Run		6/14/2022 3:36:28 PM	(GMT +02:00) W. Europe Standard Time	user_agilent	FMP1
Product Number : G2939B		Instrument	Run		6/14/2022 3:36:28 PM	(GMT +02:00) W. Europe Standard Time	user_agilent	FMP1
Name :		Instrument	Run		6/14/2022 3:36:28 PM	(GMT +02:00) W. Europe Standard Time	user_agilent	FMP1
Vendor : Agilent Technologies		Instrument	Run		6/14/2022 3:36:28 PM	(GMT +02:00) W. Europe Standard Time	user_agilent	FMP1
Serial# : DEDAE01485		Instrument	Run		6/14/2022 3:36:28 PM	(GMT +02:00) W. Europe Standard Time	user_agilent	FMP1
Firmware : C.01.069		Instrument	Run		6/14/2022 3:36:28 PM	(GMT +02:00) W. Europe Standard Time	user_agilent	FMP1
Cartridge : Electrode		Instrument	Run		6/14/2022 3:36:28 PM	(GMT +02:00) W. Europe Standard Time	user_agilent	FMP1

 © Copyright 2003 - 2018 Agilent, Inc.
